# Review on UV-Induced Cationic Frontal Polymerization of Epoxy Monomers

**DOI:** 10.3390/polym12092146

**Published:** 2020-09-20

**Authors:** Muhammad Salman Malik, Sandra Schlögl, Markus Wolfahrt, Marco Sangermano

**Affiliations:** 1 Polymer Competence Center Leoben GmbH, Rossegerstrasse 12, 8700 Leoben, Austria; muhammad.malik@pccl.at (M.S.M.); sandra.schloegl@pccl.at (S.S.); markus.wolfahrt@pccl.at (M.W.); 2 Dipartimento di Scienza Applicata e Tecnologia, Politecnico di Torino, C.so Duca degli Abruzzi 24, I-10129 Torino, Italy

**Keywords:** photoinitiator, thermal initiator, UV light, cationic polymerization, epoxy monomers, frontal polymerization

## Abstract

Ultraviolet (UV)-induced cationic frontal polymerization has emerged as a novel technique that allows rapid curing of various epoxy monomers upon UV irradiation within a few seconds. In the presence of a diaryliodonium salt photoinitiator together with a thermal radical initiator, the cationic ring opening polymerization of an epoxide monomer is auto-accelerated in the form of a self-propagating front upon UV irradiation. This hot propagating front generates the required enthalpy to sustain curing reaction throughout the resin formulation without further need for UV irradiation. This unique reaction pathway makes the cationic frontal polymerization a promising route towards the efficient curing of epoxy-based thermosetting resins and related composite structures. This review represents a comprehensive overview of the mechanism and progress of UV-induced cationic frontal polymerization of epoxy monomers that have been reported so far in literature. At the same time, this review covers important aspects on the frontal polymerization of various epoxide monomers involving the chemistry of the initiators, the effect of appropriate sensitizers, diluents and fillers.

## 1. Introduction

In the past few decades, polymer resins cured via frontal polymerization technique have been the focus of research for applications such as nanocomposites, functionally graded polymers, sensory materials, hydrogels and fibre-reinforced composites [1,2,3,4,5,6,7]. By definition, monomers that undergo frontal polymerization are able to propagate directionally in a confined reaction zone over a period of time, requiring a localized stimulus [8]. Once triggered, the polymerization front propagates in a wave-like fashion to all portions of the sample using the enthalpy of polymerization, without further need for an external stimulus. The first successful frontal polymerization was reported by Russian scientists in 1972 using methyl methacrylate as the monomer and benzoyl peroxide as thermal initiator [9]. Later, it was successfully implemented at a pilot production plant in Chernogolovka and to a polymeric material synthesis factory in Dzerzhinsk in Russia [10,11,12]. Frontal polymerization requires an external stimulus to trigger the reacting front. Based on different stimuli, frontal polymerization is classified into three categories namely thermal frontal polymerization, photo-frontal polymerization and isothermal polymerization [8].

In particular, ultraviolet (UV)-induced frontal polymerization has attracted a great number of industrial sectors for various applications due to its low operational costs, ambient temperature processing, solvent-less formulation and the freedom to have tuneable final properties of the cured polymer [13]. UV-curing is very well known for applications such as printing inks and adhesives, varnishes, protective coatings, which have been implemented on a vast scale thanks to rapid curing [13,14,15,16,17]. Photoinduced polymerizations proceed by a chain reaction mechanism involving the propagation of an active centre by the interaction with a monomer. The active centre can be a radical, a cation or more rarely an anion.

**Scheme 1:**
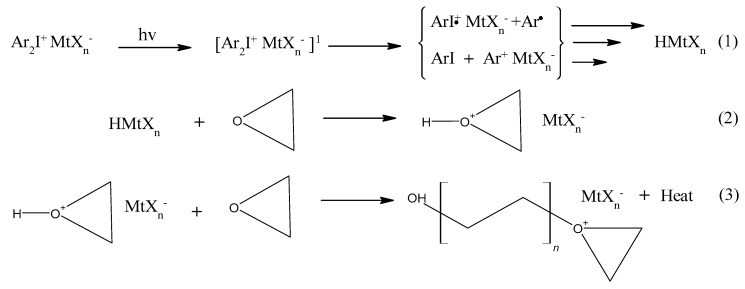
Reaction scheme for cationic ring opening frontal polymerization of oxiranes. (1) Photo-induced cleavage of iodonium salt from a singlet (S1) and/or triplet (T1) excited state, that generates cations, radical cations and free radicals ultimately producing a superacid; (2) Protonation of oxirane monomer; (3) Exothermic reaction of protonated monomer with oxiranes resulting in a polymer chain growth.

While photoinduced radical chain growth polymerization has been widely investigated and exploited from an industrial point of view, it was not until the discovery of onium salt photoinitiators by James V. Crivello in the 1970s that the first cationic photoinduced polymerization was reported [18]. Typically, onium salts, either triarylsulfonium or diaryliodonium salts, are considered as a photo-acid generator under UV-irradiation. The strong photo-acid generated is able to promote cationic ring-opening polymerization of an epoxy resin (reaction reported in Scheme 1) [18,19,20]. These highly reactive cations attack adjacent epoxide groups forming polyether in the ring opening cationic polymerization (see reaction Scheme 1) [21,22]. The Lewis acid generated in cationic polymerization sustains its reactivity with the epoxy monomers for a long time without further UV irradiation needed, as opposed to free radical polymerization where the curing reaction stops upon removal of UV irradiation [13]. This so-called “dark curing” offers the advantage of curing residual monomer and thermal post treatment for complete conversion in cationic polymerization [23]. Furthermore, cationic photopolymerizable monomers such as epoxies are less toxic and irritating in comparison to frequently used acrylates in free radical photopolymerization. Epoxide monomers cured via cationic route have lower volumetric shrinkage and also provide better matrix adhesion as opposed to free radical photopolymerized monomers [17,24,25,26]. Therefore, most of the early investigations have been focused on UV curing of epoxide monomers for inks, coatings and adhesives where they provide high polymerization rates, insensitivity to oxygen and low energy requirement [27,28,29,30,31].

However, the main limitation of UV-induced crosslinking reaction is related to the low penetration depth of UV light, which limits polymerization techniques, even cationic photopolymerization, to thin films and adhesive applications [31,32]. The first report on photoactivated cationic polymerizations to achieve self-sustained frontal polymerizations was reported by Crivello et al. [33], who showed that the local application of heat to an irradiated monomer sample results in polymerization that occurs as a front, propagating rapidly throughout the entire reaction mass [33,34,35].

**Scheme 2:**
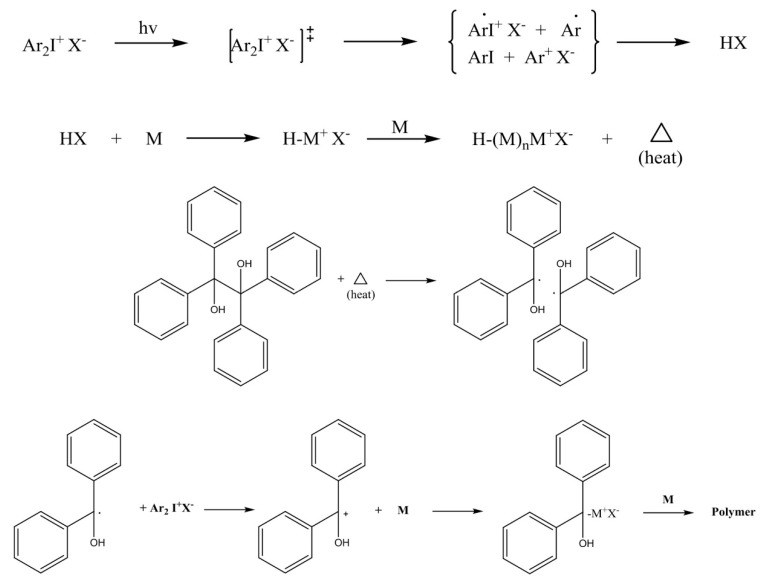
Reaction scheme illustrating the photocleavage of an iodonium salt photoinitiator together with disassociation of a thermal radical initiator to produce radical cations and free radicals respectively, during frontal polymerization of an epoxy monomer (M). Extracted from Composites Part B: Engineering, 143, M. Sangermano, A. D’Anna, C. Marro, N. Klikovits, R. Liska, UV-activated frontal polymerization of glass fibre-reinforced epoxy composites, 168–171, Copyright (2018) with permission from Elsevier.

In 2004, Mariani et al. [36] proposed the idea to combine cationic photopolymerization with frontal polymerization, as a novel method to cure epoxy monomers via UV irradiation. The cationic ring opening polymerization of epoxide monomer is a highly exothermic reaction producing 18–24 kcal/mol of energy [37]. It was shown that the frontal polymerization is started by the dissociation of a radical thermal initiator (RTI) promoted by the heat released during surface UV-induced cationic ring-opening polymerization. Subsequently, the carbon-centred radicals are oxidized to carbocations in the presence of the iodonium salt in a radical-induced cationic frontal polymerization (RICFP) mechanism [36]. In the last few years, the term RICFP has been used to define curing of epoxy and its composites specifically induced by UV light (Scheme 2) [36,38,39,40,41,42].

The purpose of the current review is to give a comprehensive overview of studies on RICFP that have been investigated in the literature so far. Since epoxy-based monomers are the most widely used components for transport and construction sectors, this review is also aimed for providing a guide for UV frontal curing of various epoxy monomers used commercially. A few authors have presented reviews on UV-activated cationic polymerization [25,43,44], but to the best of our knowledge, a review on the radical-induced cationic frontal polymerization of epoxide monomers has not been reported so far.

## 2. Chemistry of Cationic Photoinitiators Based on Diaryliodonium Salts

**Figure 1:**
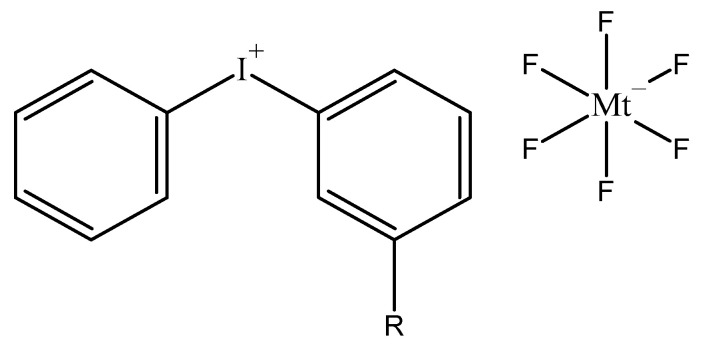
Chemical structure of a diaryliodonium salt photoinitiator. Mt represents a metallic element.

In the past, organometallic-based photoinitiators have been developed and employed for cationic photopolymerization of epoxides. Some of the photoinitiators included manganese decacarbonyl [45], complexes of ferrocene and titanium tetrachloride [46], and diazonium salts [47,48]. However, these photoinitiators had certain drawbacks that limited their applicability for epoxide curing. The Lewis acid generated from photodecomposition of a ferrocenium salt is weaker than a Brønsted acid produced from iodonium salts and required heating to complete the epoxy curing process. Moreover, ferrocenium salts suffer from poor solubility in various epoxy resins [17]. In contrast, aryldiazonium salts produce nitrogen gas in epoxy curing due to its inherent thermal instability [17]. On the contrary, epoxies cured with iodonium-based photoinitiators provide excellent adhesion to various substrates due to presence of hydroxyl groups in the growing polymer chain. Iodonium salts are colourless to pale yellow crystalline compounds having tuneable reactivity for epoxides. Figure 1 shows the structure of a diaryliodonium photoinitiator. Longer substituted alkyl chains (R groups in Figure 1) can reduce toxicity and increase solubility of iodonium salts in epoxide monomers. In addition, their reactivity can be increased by the addition of long wave sensitizers and radical generators that also prevent release of toxic gases during photocleavage, as opposed to most frequently used reactive sulfonium salts that generate toxic diphenyl sulphide gas [17,49,50]. More details about sensitizers will be discussed in the section “Effect of sensitizers”.

The cationic and anionic species in an iodonium salt photoinitiator have individual roles to play during cationic ring opening polymerization of epoxides [51]. To begin with, light absorption, quantum yield, photosensitivity and rate of photolysis of the iodonium salt is controlled by the cation. In addition, thermal stability of the iodonium salt is also governed by the type of cation present. The anion, on the other hand, controls the curing reaction in the absence of UV light and therefore determines the strength of the photogenerated Brønsted acid, and the reactivity and nature of cation radicals generated during photolysis of the iodonium salt. The rate and extent of chain growth of the epoxide monomer during cationic polymerization is also influenced by the nature of the anion [23,51]. Ideally, the anion should be highly non-nucleophilic to prevent a recombination with cations during photocleavage of the iodonium salt, which would further prevent the formation of radical cations and free radicals required to generate the superacid [51]. This relative nucleophilicity of the anion is classified into three groups of anions based on their reactivity in cationic polymerization. Strongly nucleophilic anions such as I^−^, Cl^−^, Br^−^ generate weak Brønsted acids and the ion pairs collapse to give stable covalently bonded compounds. The second type of anions, including HSO_4_^−^, ClO_4_^−^ etc., are called intermediate nucleophilic anions, while SbF_6_^−^, AsF_6_^−^, PF_6_^−^, BF_4_^−^ are termed as weakly nucleophilic anions [51]. The latter type of anions are arranged in the order of decreasing reactivity in cationic polymerization specifically for epoxide monomers [16]. These weakly nucleophilic anions used as photoinitiators slow down the polymer chain termination process during cationic polymerization. This means that the ring opening polymerization of epoxides proceeding via cationic mechanism can be seen as a “living polymerization” i.e., the polymerization proceeds until ring chain equilibria has been established, even after the irradiation has ceased [52,53].

It is also important that the emission spectrum of the irradiation source matches with the absorption properties of the iodonium salt photoinitiator so that cation and free radicals are produced during initiation [51]. The absorption wavelength of a diaryliodonium salt is very crucial while selecting the irradiation source [17]. Diaryliodonium salts typically have absorption wavelengths below 300 nm. Selected examples of such salts are diphenyliodonium hexafluorophosphate (λ_max_ = 227 nm), phenyl-*p*-octylphenyl iodonium hexafluoroantimonate (λ_max_ = 240 nm), 4-[(2-hydroxytetradecyl)oxy]phenyl] phenyliodonium hexafluoroantimonate (λ_max_ = 250 nm) [17].

In addition, a two-fold increase in light intensity doubles the rate of polymerization of epoxy containing monomers [52]. The cationic polymerization is also inhibited in the presence of bases, water and hydroxyl compounds, which may act as chain transfer agents or inhibitors [31,51].

## 3. Onium Salt Photoinitiators for Different Epoxy Monomers

In early works on the chemistry of diaryliodonium salt photoinitiators, Crivello and Lam [19], studied the effect of different parameters on photodecomposition of these salts to yield cations and cation radicals. They showed that photodecomposition of 4,4′-dimethyldiphenyliodonium hexafluoroarsenate is linearly dependant on irradiation time. However, at wavelengths higher than 300 nm, the photodecomposition of this iodonium salt was drastically reduced. It was also reported that temperature variations between 15–35 °C and oxygen environment had no significant effect on photodecomposition of the iodonium salt. In addition, there is a linear dependence of the rate of photolysis on the light intensity. Since only the nature of the cation affects photosensitivity, slight variations in absorption wavelengths gave negligible differences in the photodecomposition of methoxy, methyl, bromo and chloro substituted diphenyliodonium salts. The results also revealed that most of the diphenyliodonium salts absorb at the region of the small extinction coefficient tail of major absorption band, i.e., 237–238 nm [19].

Crivello et al. [20], performed photopolymerization of some cyclic monomers such as cyclohexene oxide, to demonstrate the effect of the nature of anion in cationic polymerization using a triphenylsulfonium salt photoinitiator. Using a high-power mercury lamp as UV source, triphenylsulfonium salts bearing each of the anions SbF_6_^−^, AsF_6_^−^, PF_6_^−^, BF_4_^−^ were investigated with cyclohexene oxide as monomer, and the conversion vs. irradiation time was monitored. The results showed that conversion of the monomers was very high with triphenylsulfonium salts bearing SbF_6_^−^ anion, followed by AsF_6_^−^ (<70%), PF_6_^−^ (<50%) and BF_4_^−^ (<5%) for cyclohexene oxide within 15 min of irradiation [20]. It was concluded that the larger the size of the anion in a photoinitiator is, the more active the propagating cationic species will be in the ring opening polymerization [20]. In another study, Crivello et al. [27] also demonstrated the effect of photoinitiator concentration, wavelength and intensity of irradiating light, effect of temperature and water vapour on the photopolymerization of cyclohexene oxide. To sum up, triphenyl sulfonium salts added with 1.5% molar concentration and high UV intensity gave maximum cure rate within 20 s of irradiation. In addition, temperatures above 30 °C and amounts of water greater than 1–2% by weight increase the time to reach maximum epoxy curing and irradiation time, respectively [27]. In a study on photoinitiated cationic polymerization of various silicon-containing epoxies, Crivello and Lee [54] showed that diaryliodonium salt containing a SbF_6_^−^ anion gives higher polymerization rate and exothermicity than PF_6_^−^ in the cationic ring opening polymerization of silicon-containing cycloaliphatic epoxy [54].

**Figure 2:**
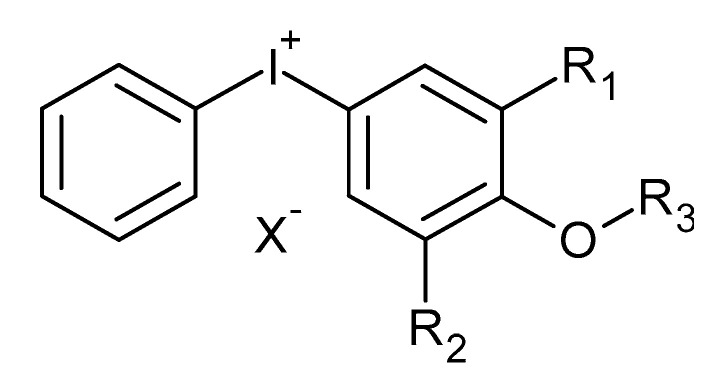
Chemical structure of a 4-alkoxy substituted photoinitiator. Redrawn from J. V. Crivello and J. L. Lee, Journal of Polymer Science Part A: Polymer Chemistry, 1989, 27, 3951–3968, John Wiley and Sons, Copyright 1989 John Wiley & Sons, Inc.

In their study, Crivello et al. [49] investigated the properties of 4-alkoxy-substituted diaryliodonium salt photoinitiators in cationic polymerization of heterocyclic monomers. The general structure of the 4-alkoxy iodonium salt photoinitiator is shown in Figure 2. The R_3_ group in this structure was varied between C_8_ to C_18_ in order to characterize the effect on melting point, solubility, spectral characteristics, toxicology and photosensitivity of the iodonium salt photoinitiator. It was found that the melting point increases linearly with a higher number of carbon atoms and all photoinitiators having alkoxy substitution > C_8_ were soluble in toluene, styrene and some epoxidized organic compounds. The absorption wavelengths for all these photoinitiators varied between 245–247 nm only, i.e., in the short wavelength region of UV spectrum. This slight variation in absorption wavelength for substituted alkyl groups was also reported for diaryliodonium salt photoinitiators in a previous study [19]. The toxicity (lethal oral dose) for C_8_ to C_18_ was also lower in comparison to methoxy substituted salts. In addition, the alkoxy-substituted diaryliodonium salts gave higher UV cure rates in comparison to alkyl substituted diaryliodonium salts. By increasing the concentration of alkoxy-substituted salt up to 3%, the photo efficiency could be improved which enabled a cure with even low power lamps. These salts could also be photosensitized with various species to further increase their photosensitivity [49].

Castellanos et al. [55] synthesized a novel diaryliodonium salt photoinitiator based on tetrakis (pentafluorophenyl) borate anion and performed thin film cationic polymerization with a cyclohexene oxide epoxy and silicone epoxy monomers. The group reported that the rate of propagation of tetrakis (pentafluorophenyl) borate anion is twice as high as the rate of propagation of hexafluoroantimonate anion in the cationic polymerization of dicyclohexene oxide epoxy and 2.5 times higher in the case of epoxy silicone monomer. It was also found experimentally that the mechanism of photolysis for tetrakis (pentafluorophenyl) borate anion type photoinitiator and its relative products is similar to hexafluoroantimonate type iodonium salt. This novel anion iodonium salt photoinitiator also proved to be resistant to humidity with several days pot life in epoxy silicone monomer [55].

In the first study on radical-induced cationic frontal polymerization conducted by Mariani et al. [36], {4-[(2-hydroxytetradecyl) oxy] phenyl} phenyliodonium hexafluoroantimoniate (HOPH) as photoinitiator was tested in various concentrations with bis-cycloaliphatic epoxide (CE) and dibenzoyl peroxide (BPO) as thermal initiator. It was found that the minimum ratio of HOPH and BPO required for a self-sustaining front is 1 mol/mol. With a constant 1 mol/mol HOPH/BPO molar concentration, results of RICFP experiments proved that an increase in HOPH with respect to CE concentration increased the frontal velocity up to a molar ratio of 2, while a maximum front temperature of 256 °C was observed at a molar ratio of 1.35. At a higher molar ratio of 3 mol/mol % of HOPH/CE, the maximum front temperature decreased to 232 °C. Results from Differential Scanning Calorimetry (DSC) plots showed that both initiators are necessary to allow curing also in the bulk of sample where light penetration is hindered [36].

In the studies conducted by Bomze et al. [38] on radical-induced cationic frontal polymerization of various epoxies, two commercially available photoinitiators were tested for their applicability in RICFP of bisphenol A diglycidyl ether (BADGE). It was found that RICFP with equimolar ratio of a thermal initiator and diphenyliodonium tetrakis (pentafluorophenyl) borate as photoinitiator gave a successful self-sustaining polymerization front. Comparing this photoinitiator with a triphenylsulfonium salt, it was found that the latter did not generate a front even at 5 wt% in the resin. This was explained by the lower redox potential of sulfonium salts compared to the diaryliodonium ones [38]. In another study, Bomze et al. [39] investigated the effect of variable concentration of *p*-(octyloxyphenyl)phenyliodonium hexafluoroantimonate (PAG) on storage stability, thermal and frontal polymerization properties of a formulation consisting of BADGE and tetraphenyl ethanediol (TPED). Results confirmed the good storage stability of a formulation of BADGE with 1 mol/mol molar ratio of PAG/TPED giving only slight variation in viscosity for 28 days of storage. In addition, the maximum front velocity and temperature were also unaffected by the storage time. It was also shown that a formulation of BADGE with 1 mol % PAG and increasing TPED concentration reduces the maximum front temperature. In contrast, with 2 mol % PAG the front temperature reaches a maximum of 215 °C up to 1 mol % TPED but then decreases with increasing concentration of TPED. The glass transition temperature (T_g_) of BADGE formulation changes only slightly with the change in molar ratio of PAG:TPED. In particular, 168 and 160 °C were obtained at 2:1 and 1:1 molar ratio of PAG:TPED, respectively. It should be further noted that a higher T_g_ was achieved with frontally cured epoxy resins than with only photocured and thermally cured counterparts [39].

Klikovits et al. [56] synthesized novel photoinitiators including diphenyliodonium tetrakis(perfluoro-t-butyloxy)aluminate (I-Al) and tris(4-((4-acetylphenyl)thio)phenyl)sulfonium tetrakis(perfluoro-t-butyloxy)aluminate (S-Al) that exhibited a higher photo efficiency than the diphenyliodonium hexafluoroantimonate (I-Sb) and diphenyliodonium tetrakis(perfluorophenyl) borate (I-B) photoinitiators in the cationic ring opening polymerization of epoxide monomers. This weakly coordinating anion (WCA) based on the tetrakis(perfluoro-*t*-butyloxy)aluminate anion gave a stronger Brønsted acid than the conventional antimonate and borate anions. The novel photoinitiators I-Al and S-Al were tested with BADGE and 1,4-bis[(3-ethyl-3-oxetanylmethoxy) methyl] benzene (BOB) and compared with their respective SbF_6_ and (BC_6_F_5_)_4_^−^ anions. It was found that both diphenyliodonium and triarylsulfonium salts based on Al anion exhibited higher polymerization rates and monomer conversion than their corresponding SbF_6_^-^ and (BC_6_F_5_)_4_^−^ anions [56]. Knaack et al. [40] also tested an epoxy formulation consisting of BADGE, I-Al and TPED for its effect on frontal polymerization properties. It was found that a minimum of 0.015 mol % of I-Al with 1 mol % TPED is sufficient to achieve a self-sustaining front. Front velocity and temperature increase up to 1 mol % of I-Al reaching a maximum of 10 cm min^−1^ velocity with a front temperature of 210 °C. Comparing the front starting time of I-Al with I-Sb, the group showed that front starting time decreases with the content of I-Al whereas for I-Sb, no front was possible below 1 mol %. The onset temperature of thermal curing for the BADGE formulation with 0.015 mol % I-Al was only slightly lower than with 1 mol % I-Sb. Results from storage stability tests conducted on a formulation of BADGE with 0.015 and 1 mol % I-Al showed no significant change in viscosity and corresponding frontal velocity and frontal temperature for 28 days of storage. More than 99% conversion for BADGE was achieved with a formulation of 1 mol % I-Al, while with I-Sb a 97% conversion was obtained. The T_g_ of the cured formulation with 0.5 mol % I-Al was almost similar to the T_g_ for the BADGE formulation cured with 1 mol % I-Sb. The group also investigated the effect of variable mould depth on the frontal polymerization of BADGE using I-Al and I-Sb photoinitiators by measuring the minimum layer thickness of the cured polymer. The results showed a three times lower minimal layer thickness of the cured formulation containing I-Al compared to I-Sb at 1 mol % with increasing concentration of thermal radical initiator [40].

Wang et al. [57] reported a novel combination of a photoinitiator based on diaryliodonium salt and silanes that gave adjustable gel time of polymerization as well as excellent storage stability for bisepoxides. This unique formulation provided the advantage of multi-mechanism curing, which includes redox free radical, free radical and thermal/UV-induced cationic polymerization. They proved the positive influence of silane groups in cationic photo polymerization of epoxy, where Si-H groups where able to participate in the initiation and promotion of epoxy polymerization [57].

### Effect of Sensitizers

Sensitization is required in formulations containing a photoinitiator that is unable to absorb in the spectral region of the provided irradiation source. Upon absorption of light, the sensitizer is photoexcited and transfers the energy to the photoinitiator, which reacts by cleavage and release of reactive species. Different types of sensitizers have been classified by Fouassier [31] and Green [17]. Photosensitization of onium salts can be divided into three mechanisms: (1) oxidation of certain photolysable compounds by photoinitiator to form free radicals, (2) electron transfer between a photoexcited molecule and onium salt to give sensitizer radical cations and (3) electronically excited charge-transfer complex formed between an aromatic pyridinium and onium salt to generate aromatic radical cations [58,59,60].

Diaryliodonium salts cannot photo-decompose under visible light (400–800 nm) and require sensitization to generate radicals and cations during initiation. Different organic dyes were investigated by Crivello et al. [21] for their sensitization effect on 4,4′-dimethyldiphenyliodonium hexafluoroarsenate. It was shown that sensitized photopolymerization of cyclohexene oxide with sensitizers benzoflavin and acridine orange is possible with visible light within few minutes of irradiation. 4,4′-dimethyldiphenyliodonium hexafluoroarsenate sensitized with benzoflavin and acridine orange gives a photodecomposition of 60 and 46%, respectively, and there exists a maximum concentration limit for sensitizers that could effectively photolyse the diphenyliodonium salts. The λ_max_ for benzoflavin and acridine orange was found to be 460 and 539 nm, respectively, in the cationic photopolymerization of cyclohexene oxide. Dye sensitized photopolymerization of cyclohexene oxide and epichlorohydrin was also compared using 4,4′-di-*tert*-butyldiphenyliodonium hexafluoroarsenate as photoinitiator. A 35% conversion was achieved within 900 s of irradiation for cyclohexene oxide while due to sluggish reactivity of epichlorohydrin, conversion was achieved after 2.5 h of irradiation [21].

Crivello and Lam [54], investigated the photosensitivity of 2-chlorothioxanthone (λ_max_ = 386 nm) using a diphenyliodonium-based photoinitiator in the cationic polymerization of siloxane-functional epoxy monomers. Significant increase in polymerization rate was observed using 0.05 mol % of this sensitizer with 0.5 mol % of the diphenyliodonoium salt [54]. In 1994, Yagci and Schnabel [61] demonstrated the photosensitivity of pyridinium and quinolinium salts in the cationic photopolymerization of epoxy and vinyl ether monomers. They showed that reactive cations for the chain initiation can be formed either by direct photolysis of the pyridinium ions or by electron transfer from photogenerated free radicals or from photoexcited sensitizers to pyridinium salts [61]. A detailed study on the pyridinium and benzyl salts sensitization mechanism was also published by Yagci and Endo [62]. Bi and Neckers [63] introduced a novel combination of xanthene dye and an aromatic amine combined with diaryliodonium salt photoinitiator that was able to initiate the cationic ring opening polymerization of various epoxide monomers under visible light (>520 nm). The xanthene dye absorbs visible light to produce free radicals, which are oxidized by the diaryliodonium salt and reduced by aromatic amine to form α-amino radical, that is further oxidized by iodonium salt to generate α-amino carbocation, ultimately initiating the cationic polymerization. The xanthene dye provided a range of absorption maxima in order to have adjustable photosensitivity of the epoxy and iodonium formulation. The results also showed different irradiation times for a range of epoxide monomers. For example, bisphenol A diglycidyl ether required <300 min of irradiation, while cyclohexene oxide only 10 min. However, this initiator system cannot polymerize bis-cycloaliphatic epoxy monomers [63].

Yagci et al. [58] introduced a ternary system consisting of an alkyl halide, a dimanganese decacarbonyl, and a diaryliodonium salt capable of performing cationic polymerization of bisepoxides under visible light (435.8 nm). Free radical-promoted cationic polymerization of cyclohexene oxide was demonstrated using this ternary system. They showed that the oxidation of carbon-centred radicals to yield reactive cations depends on the polarity of alkyl halide and the redox potentials of radicals and iodonium salt [58]. Chen et al. [64] reported high photosensitization efficiency of carbazole derivatives (λ_max_ = 365 nm) in the cationic photopolymerization of bis-cycloaliphatic epoxy monomer using diaryliodonium salt photoinitiators. These electron transfer photosensitizers provided either singlet or triplet excited photosensitization depending on the substituent groups in the carbazole compounds [64].

In 2000, Hua et al. [65] showed a marked influence of *N*-vinylcarbazole as a photosensitizer and as an accelerator in the cationic ring opening polymerization of various cyclic and aliphatic epoxide monomers using diaryliodonium salt photoinitiators. This photosensitizer follows an electron transfer photosensitization mechanism that involves a redox reaction between the excited carbazole and the onium salt. At high concentration, the presence of a photoinitiated free-radical chain reaction results in the generation of a large number of cationic species capable of initiating both *N*-vinylcarbazole and epoxide ring-opening polymerizations [65]. In another study, Hua and Crivello [66] reported enhanced UV spectral absorption of the polymer of *N*-vinylcarbazole, proving its remarkable capability as photosensitizer in the cationic ring opening polymerization of epoxy monomers.

Crivello et al. [67] developed a novel sensitized cationic curing mechanism for multifunctional epoxy monomers by combining direct and free radical-induced decomposition of diaryliodonium salts. They showed that camphorquinone upon photoexcitation generates radical species by abstracting a hydrogen from monomer that induces the free radical decomposition of the diaryliodonium salt. Different cyclic epoxide monomers were tested, and it was found that bis-cycloaliphatic epoxy was the most reactive epoxy, stabilizing the free radical and cationic species that are formed. The results showed that aryl ketones, 1,2-diketones and quinones are effective photosensitizers, allowing cationic curing of epoxies under visible and long-wavelength UV [67].

In early 2002, Hua et al. [68] introduced a novel electron transfer photosensitizer based on polynuclear aromatic hydrocarbons modified with hydroxymethyl groups. These hydroxyl-methylated compounds such as 9-anthracenemethanol, pyrene, 3-perylenemethanol were able to participate in the cationic ring opening polymerization of 4-vinylcyclohexene diepoxide through the activated monomer mechanism due to the presence of hydroxyl groups, hence increasing the polymerization rate. These hydroxyl groups enhance solubility of the photosensitizer in the epoxy monomer and also reduce toxicity of the photosensitizer by forming covalent bonds with terminating ether groups in the polymer chain [68]. Gomurashvili and Crivello [69] reported dual property of phenothiazines as photosensitizers as well as co-monomers in the cationic ring opening polymerization of various epoxide monomers. These polymerizable photosensitizers also possessed an absorption maximum between 254–333 nm and were able to speed up the cationic ring opening polymerization of various epoxides [70].

In another study, the photosensitivity of an electron transfer photosensitizer based on pyrene derivatives in the cationic ring opening polymerization of epoxide monomers was demonstrated by Crivello and Jiang [71]. A diaryliodonium salt photoinitiator was sensitized with pyrene derivative and was found to give higher polymerization rates and monomer conversion for cyclo-epoxy and bis-cycloaliphatic epoxy monomers than with non-sensitized formulations [71].

In 2003, Crivello and Jang [72] prepared several anthracene compounds as electron transfer photosensitizers for cationic ring opening polymerization of various epoxide monomers. These anthracene compounds possessed a range of absorption maximum between 217–403 nm, among which 9,10-dialkoxyanthracene possessed the highest photosensitization efficiency. It was shown that anthracene derivatives with electron-withdrawing groups are not effective photosensitizers [72].

Different pigments (red, white and blue) were found to decrease the photoreactivity in the cationic polymerization of a siloxane and cyclohexene oxide using a diaryliodonium salt as photoinitiator [73]. A novel approach to sensitize epoxide compounds was introduced by Crivello et al. [74], in which electron transfer photosensitizers such as *N*-vinyl carbazole, *N*-allyl carbazole and 9-vinyl anthracene, were covalently bound directly to the structure of the a polymeric photoinitiator. These polymer-bound sensitizers were found to accelerate the rate of polymerization in the cationic ring opening photopolymerization of difunctional epoxies as well as increase the solubility in various organic solvents [74].

In 2005, Crivello and Bulut [75] reported curcumin as a naturally occurring long-wavelength (340–535 nm) photosensitizer capable to perform cationic ring opening polymerization of various epoxides. This electron transfer photosensitizer was found to be soluble in various monomers and able to initiate upon exposure with LEDs and sunlight. Due to its slightly acidic nature it did not retard cationic photopolymerization [75]. Subsequently, in 2006, the group demonstrated the broad UV and visible light absorption property of curcumin in the cationic ring opening polymerization of bio-based epoxy monomers [76]. Photopolymerization of synthetic oxirane and oxetane monomers was successfully performed upon exposure with 355–460 nm using curcumin-sensitized diaryliodonium salt photoinitiator. In addition, glass fibres impregnated with curcumin sensitized linseed oil with epoxy groups and *Veronica galamensis* oil was successfully fabricated and photopolymerized under direct sunlight [76].

Crivello [77] performed photopolymerization of neopentyl glycol diglycidyl ether in the presence and absence of 9,10-di-*n*-butoxyanthracene as a photosensitizer. In the presence of the photosensitizer, the polymerization begins slowly upon UV exposure, with smaller induction period in comparison to non-sensitized formulations. The polymerization is auto-accelerated, as the temperature of the sample increases. The temperature–time curves showed that the profiles of sensitized and non-sensitized formulations are very similar and both formulations reach essentially the same maximum temperature (159 vs. 156 °C) [77]. In another study, Crivello [78] investigated a three-component system consisting of camphorquinone as sensitizer, a diaryliodonium salt as photoinitiator, and benzoyl alcohol. The epoxy curing followed a free radical chain-promoted cationic polymerization scheme induced by visible light, in which the latter photoexcites camphorquinone. Benzoyl alcohol donates a proton to generate a radical that reacts with diaryliodonium salt, producing a Brønsted acid. Crivello also studied the various experimental parameters that affect free radical chain-promoted cationic ring opening polymerization of limonene dioxide epoxy using optical pyrometry (monitoring temperature vs. irradiation time). A combination of limonene dioxide, camphorquinone, methoxybenzyl alcohol and photoinitiator based on tetrakis (pentafluorophenyl) borate anion cured the epoxy under visible light within 15 s with a highest temperature of more than 165 °C. It was found that cyclohexene oxide and 1,2-epoxyoctane displayed higher reactivities than *n*-butyl glycidyl ether. Crivello also fabricated a flat composite structure composed of four layers of glass cloth impregnated with a formulation consisting of dicycloepoxycarboxylate, 2.5% 4-*n*-(octyloxyphenyl) phenyliodonoium hexafluoroantimonate, 1.5% camphorquinone, and 20% methoxybenzyl alcohol. The assembled composite was exposed to direct sunlight for 3 min, during which fast, highly exothermic polymerization took place giving a completely dry, rigid and clear crosslinked laminate [78]. Along the same lines, a study on free radical chain-promoted cationic polymerization induced by visible light for various epoxide monomers was also done using titanium complex free radical photoinitiator instead of camphorquinone [79]. Similarly, another three-component system based on ethyl-4-dimethyl aminobenzoate, camphorquinone and a diaryliodonium salt showed epoxy curing upon visible light irradiation [80].

Pure BADGE has a sluggish reactivity in cationic polymerization. Moreover, sensitization has to be compromised with monomer conversion to achieve a successful polymerization front. In this regard, Liu et al. [81] reported cationic polymerization of BADGE with an iodonium salt photoinitiator sensitized with dihydroanthracene in 1:1 molar ratio quantity. They used light with an irradiation wavelength of 385 nm and found that heating the formulation from 30 to 60 °C increases the epoxy conversion by 25%. In addition, reactive diluents such as *n*-hexanol and dodecyl vinyl ether or the addition of a free radical initiator can also increase the rate of polymerization of BADGE [81]. Telitel et al. [82], reported chromophores based on boron–dipyrromethene (BODIPY) and boranil derivatives that were found to sensitize iodonium salt photoinitiator giving maximum absorption wavelengths in the range 400–600 nm and different oxidation potentials. Cationic polymerization of a bis-cycloaliphatic epoxy formulation sensitized with boranil and BODIPY derivatives was achieved at absorption wavelengths of 457 nm and 473 nm, respectively, with more than 60% monomer conversion upon 200 s of irradiation [82].

Recently, carbazole derivatives have also gained attention for their broad near-UV and visible light absorption [83,84,85]. In one study, Xiao et al. [86] found that *N*-vinylcarbazole possesses tri-functional properties, i.e., as a photoinitiator, additive and a monomer, that performs curing under the household UV-light emitting diode (LED) wavelength of 392 nm. In the case of epoxy, combination of *N*-vinylcarbazole and iodonium salt upon UV-LED irradiation generates cations based on *N*-vinylcarbazole that subsequently react with epoxy monomers to initiate the cationic ring opening polymerization. The group reported about 70% of monomer conversion within 1 min of UV irradiation [86].

Bomze et al. [39], investigated the effect of 2-isopropylthioxanthanone (ITX) with a bis-4-(methoxybenzoyl) diethylgermanium (Ivocerin) radical photoinitiator and perylene on photofrontal polymerization of BADGE. They showed that an optimum concentration of ITX is crucial to prevent shielding effect during sensitization of the diaryliodonium salt. A concentration of 0.2 mol % ITX sensitizer allows curing greater than UV light wavelengths without affecting frontal parameters in the RICFP of BADGE [39]. In another study, sensitization of two diaryliodonium salt photoinitiators was compared using ITX upon light exposure within a wavelength range of 400–500 nm to analyse whether photopolymerization of BADGE was possible in the visible light spectral region [56]. It was found that sensitization of diaryliodonium salt based on tetrakis (perfluoro-*t*-butyloxy)aluminate anion enabled the polymerization of a BADGE formulation upon light exposure at wavelengths between 400 and 500 nm. A higher polymerization rate and monomer conversion were found compared to the formulation consisting of a sensitized diaryliodonium hexafluoroantimonate photoinitiator [56].

**Figure 3:**
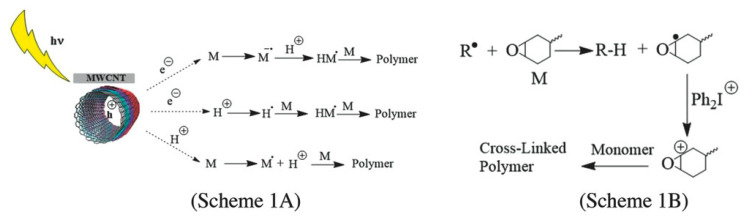
(**A**) Radical polymerization upon visible light irradiation on multi walled carbon nanotubes. (**B**) Oxidation of these radicals followed by hydrogen abstraction. Extracted from Marco Sangermano, Damian Rodriguez, Monica C. Gonzalez, Enzo Laurenti, and Yusuf Yagci, Macromolecular Rapid Communications, 2018, 39, 1800250, Copyright 2018 WILEY-VCH Verlag GmbH & Co. KGaA, Weinheim, with permission from John Wiley and Sons.

In their studies, Sangermano et al. [87] demonstrated the effect of multi walled carbon nanotubes (MWCNT) as free radical generator induced by visible light for the cationic polymerization of bis-cycloaliphatic epoxy using diaryliodonium salt. The mechanism involves a cationic polymerization of epoxides through visible light generated electrons and holes from MWCNTs (Figure 3A), followed by electron transfer reactions with the iodonium salt (Figure 3B) [87].

The incorporation of photoredox catalysts have also been considered in the cationic polymerization of epoxies where the latter provides the advantage of cheap and safe irradiation source such as LEDs, sunlight etc. [88,89,90,91,92]. In addition, several new compounds have also shown broad visible light absorption in the cationic polymerization of epoxides, such as azahelicenes [93], rubrene [94], *N*-[2-(dimethylamino)ethyl]-1,8-naphthalimide [95], spiroxanthene [96], polyoxometalate [97], nanoparticles and ferrocenium salts [98], etc.

Several photoinitiator systems based on charge transfer complex have also been reported in the cationic polymerization of epoxides [99,100,101,102]. In one study, Garra et al. [103] reported a charge transfer complex (CTC) based on mixture of *N*-phenylglycine and iodonium salt (NPG-Iod)_CTC_ that proved to be an efficient photoinitiating system in cationic polymerization of bis-cycloaliphatic epoxy monomer, enabling a curing under LED irradiation with a wavelength of 405 nm. The resulting epoxy network was clear, and thicker composites (about 9 cm) were fabricated using (NPF-Iod)_CTC_ epoxy formulation [103].

## 4. Chemistry of Thermal Radical Initiators

**Figure 4:**
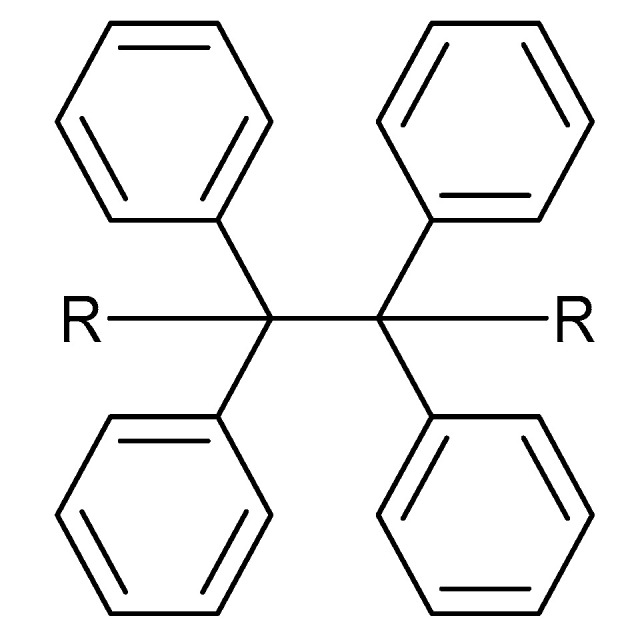
General chemical structure of a carbon–carbon labile thermal radical initiator.

Mariani et al. [36] were the first to study the effect of a thermal radical initiator on the photofrontal polymerization of a bis-cycloaliphatic epoxy (CE). They found that dibenzoyl peroxide (BPO) as thermal radical initiator gave a maximum front velocity of 6 cm min^−1^ with a maximum front temperature of 300 °C at 0.67 mol % BPO/CE. They demonstrated the importance of the combined effect of both photoinitiator and thermal radical initiator at equimolar ratio in achieving maximum epoxy conversion [36]. A detailed investigation on the structure–property relationship of thermal radical initiator on the curing of epoxy resins was done by Bomze et al. [38]. They showed that generation of thermal radicals in cationic polymerization of epoxy monomers is dependent on the substituted R groups in carbon–carbon (C‒C) labile compounds (Figure 4). They also found that apart from bulky (e.g., phenyl) groups present in C‒C labile compounds, presence of oxygen atom in the R group is also necessary to give a highly reactive thermal radical initiator capable of generating stable radical species [38].

Zhou et al. [104] tested a thermal initiator based on boron trifluoride amine complex in the cationic UV-induced frontal polymerization of a bis-cycloaliphatic epoxy with a triaryl sulfonium salt photoinitiator. Front velocity and front temperature increased with rising concentration of the thermal initiator (from 3 up to 5 wt. %). They showed that a propagating front angle is formed, which is directly proportional to the concentration of the thermal initiator, and it can be controlled also by the addition of an inorganic filler. Thermal and mechanical tests confirmed higher curing degree of the epoxy monomer than the thermally cured resin with high values of T_g_ directly proportional to an increasing thermal initiator concentration. They also showed that almost complete epoxy conversion can be achieved with a high concentration of thermal radical initiator without the need for a post curing [104]. Analogous to this study, Knaack et al. [40] showed in the frontal curing of a BADGE formulation that the presence of a thermal radical initiator reduces the onset temperature of thermal curing from 190 to 110 °C. In addition, an increase in concentration of tetraphenyl ethanediol (TPED) (>1 mol %) only slightly decreases the thermal onset temperature of the curing [40].

## 5. UV-Induced Cationic Frontal Polymerization of Different Epoxies

Studies on the cationic UV-induced frontal polymerization were not reported until in 2004, when Mariani et al. [36] demonstrated the frontal polymerization of a bis-cycloaliphatic epoxy using a photo and thermal radical initiator. However, before the inception of the concept of frontal photopolymerization, the group of Crivello published several important studies that were focused on cationic photopolymerization of various in-house synthesized epoxy monomers requiring prolonged UV irradiation of the entire formulation. These studies were mostly focused on the reactivity of epoxy monomers based on their chemical structures, effect of various functional groups and experimental parameters such as UV intensity, temperature and so on with the help of FT-IR spectroscopy and photocalorimetry [105,106,107,108,109,110,111,112,113,114].

Among the various epoxy monomers investigated in the past, particularly the cyclo-aliphatic epoxy monomer 3,4-epoxycyclohexylmethyl-3′,4′-epoxycyclohexane has been employed commercially under various trade names. Crivello and Varlemann [115] investigated the reactivity of 3,4-epoxycyclohexylmethyl-3′,4′-epoxycyclohexane. They demonstrated the retarding effect of ester carbonyl and ether groups present within 3,4-epoxycyclohexylmethyl-3′,4′-epoxycyclohexane in photoinitiated cationic polymerization. Dioxacarbenium ions are produced as a result of interaction with oxonium ions and possess high steric hindrance and low reactivity. Based on the structure of 3,4-epoxycyclohexylmethyl-3′,4′-epoxycyclohexane, possible precursors were prepared and investigated for their reactivity using real-time infrared spectroscopy. It was found that cyclohexene oxide and hydroxy cyclohexene oxide were the most reactive epoxides followed by ether and ester substituted cyclohexene oxide [115,116,117]. In another analogous study, Crivello and Liu [118] investigated the reactivity of some in-house prepared epoxy alcohol monomers in the cationic ring opening polymerization using real-time infrared spectroscopy. They showed that these hydroxy epoxies undergo faster kinetics via the activated monomer mechanism than their corresponding methoxy monomers. Their study proved that monomers bearing both hydroxy and epoxycyclohexane groups are low viscosity transparent liquids that can accelerate the rate of cationic polymerization of various epoxy compounds, giving hyperbranched polymers [118].

**Figure 5:**
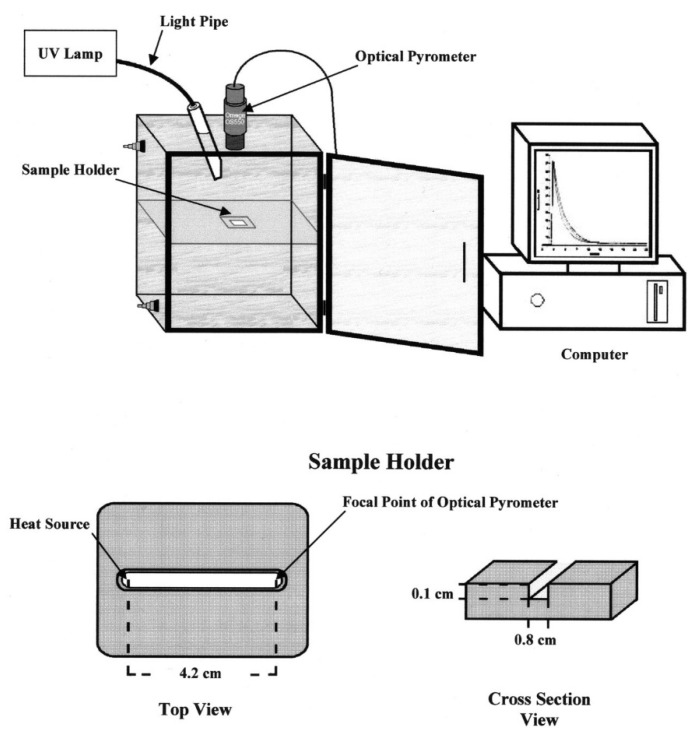
Apparatus used by Crivello et al. for studying photopolymerization of various monomers. Extracted from J. V. Crivello, B. Falk, M. R. Zonca, JR., Journal of Polymer Science Part A: Polymer Chemistry, Vol. 42, 1630–1646, Copyright 2004 Wiley Periodicals, Inc., with permission from John Wiley and Sons.

In 2003, Falk et al. [73] introduced optical pyrometry as a versatile tool for studying the effect of monomer structure, initiator concentrations and experimental parameters such as light intensity and temperature, on the cationic photopolymerization of various epoxies (Figure 5). Optical pyrometry allowed monitoring of reaction temperatures during the cationic photopolymerization of various epoxy monomers and was also combined with real-time FT-IR spectroscopy to give monomer conversion profiles. Results showed that 4-vinylcyclohexene dioxide exhibited the highest photoreactivity followed by bis1,2(2-(3,4-epoxycyclohexylethyl))-1,1,2,2-tetramethyldisiloxane and 3,4-epoxycyclohexylmethyl-3′,4′-epoxycyclohexane (ERL 4221-E). Studies on the photopolymerization of PC-1000 showed that diaryliodonium-based photoinitiator gives the highest photoreactivity, with a direct relation between photoinitiator concentration and maximum exothermic temperature up to concentration of 1 mol %. In addition, oxetanes were found to have long induction periods, which decreased with increasing temperatures. More importantly, the group found that glass and poly methyl methacrylate substrates provided successful polymerization due to their low thermal conductivity in comparison to aluminium substrate. A pronounced effect of cyclohexene oxide (CHO) was observed with oxetane, where the former reduces induction time and completes the cationic polymerization within 80 s of irradiation owing to its very high reactivity. In contrast, benzylic alcohols have also proven to be effective accelerators in the cationic polymerization of ERL 4221-E [73].

In another study, Crivello et al. [33] reported the frontal polymerization of various oxetanes, alkyl glycidyl ether and bis-cycloaliphatic epoxy monomers initiated by UV triggering using optical pyrometry. They observed frontal polymerization behaviour of oxetane monomers, which were triggered by UV and heating. However, due to high reactivity of cycloaliphatic epoxides, UV triggering was enough to complete the cationic polymerization of these monomers specifically. They found that 1,4-butanediol diglycidyl ether (BDGE) and neopentyl glycol diglycidyl ether (NDGE) frontally polymerize under UV irradiation for 6 min, reaching a very high front temperature of 250 °C. The resultant polymer was darker due to decomposition occurring at such a high temperature. On the contrary, aryl glycidyl ethers such as phenyl glycidyl ether and 4-cresyl glycidyl ether did not undergo frontal polymerization due to their inherent sluggish reactivities. These studies revealed that frontal polymerization is a characteristic of only those monomers that possess ether oxygen atoms, which are able to stabilize the protonated oxonium ion species formed during acid and monomer reaction. Due to the presence of aromatic groups in aryl glycidyl ether and 4-cresyl glycidyl ether, the oxonium ion interacts with the aromatic group, thereby reducing the stability of the protonated oxonium ion. In addition, because of similar basicity of ether oxygen and oxirane atoms in aliphatic and arylalkyl glycidyl ethers, frontal polymerization is not affected [33].

In 2004, Crivello et al. [119] studied the effect of monomer structures on the cationic photopolymerization of various epoxide and oxetane monomers. It was shown that an appreciable induction period is necessary for epoxy monomer to post cure, after irradiation is terminated. The photopolymerization of 4-vinyl-1-cyclohexene dioxide (VCHDO) reached a maximum front temperature of 270 °C while CHO reached up to 63 °C owing to the lower epoxy equivalent weight of VCHDO than CHO.

**Figure 6:**
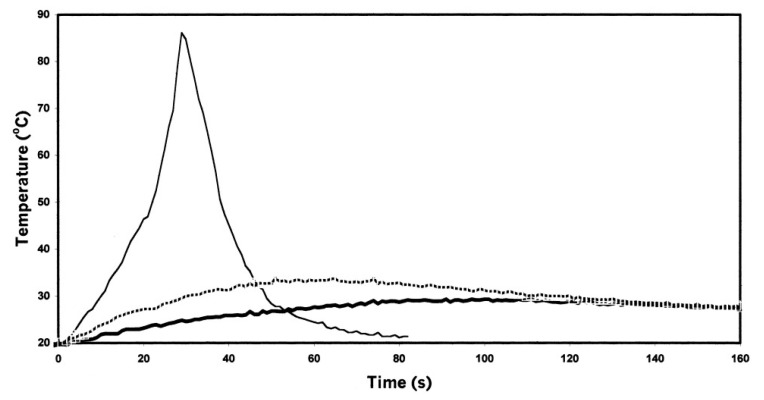
Effect of cyclohexene oxide (CHO) on the polymerization temperature and irradiation time in cationic photopolymerization of benzyl glycidyl ether (BGE). Extracted from J. V. Crivello, B. Falk, M. R. Zonca, JR., Journal of Applied Polymer Science, Vol. 92, 3303–3319, Copyright 2004 Wiley Periodicals, Inc., with permission from John Wiley and Sons.

**Figure 7:**
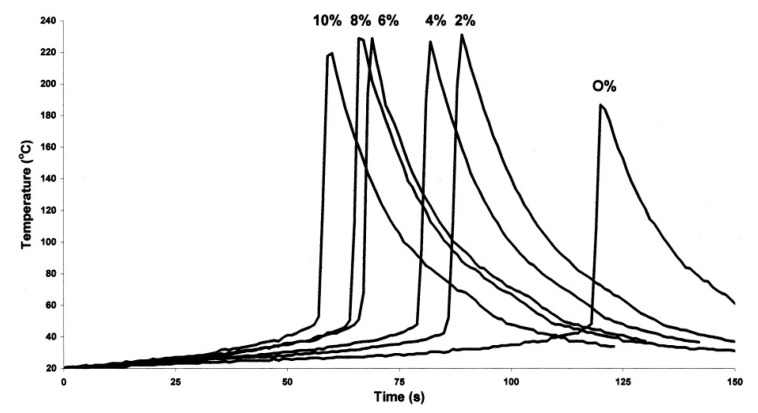
Effect of 4-vinyl-1-cyclohexene dioxide (VCHDO) on the polymerization temperature and irradiation time in cationic photopolymerization of neopentylglycol diglycidyl ether (NPGE). Extracted from J. V. Crivello, B. Falk, M. R. Zonca, JR., Journal of Applied Polymer Science, 92, 3303–3319, Copyright 2004 Wiley Periodicals, Inc., with permission from John Wiley and Sons.

With increasing chain length (1 to 5) it was shown that the maximum polymerization temperature decreased due to a decrease in the density of epoxycyclohexyl groups present in the silicone epoxy monomer. They also found that CHO and VCHDO comprised a higher reactivity with respect to benzyl glycidyl ether (BGE) and neopentylglycol diglycidyl ether (NPGE). Figure 6 and Figure 7 show the increase in polymerization temperature and irradiation time with the addition of reactive diluents (CHO and VCHDO) in the cationic ring opening polymerization of BGE and NPGE. Figure 7 also reveals that NPGE suffers from a lower reactivity in cationic polymerization due to longer induction period than BGE, which is a characteristic feature of this epoxy monomer. On the contrary, ERL 4221-E has a shorter induction time but a sluggish polymerization. The authors also described a possible effect of relative humidity on the cationic ring opening polymerization of epoxy monomers. In the presence of water, the epoxy protonation is taken over by water, subsequently generating a diol. Water is removed during the reaction to produce alcohols that ultimately react with protonated epoxides to form ether linkages via activated monomer mechanism. As a consequence, there is less steric hindrance for epoxide monomers to form longer linkages under high relative humidity as compared to the conventional epoxide linkages during cationic ring opening polymerization [119].

**Figure 8:**
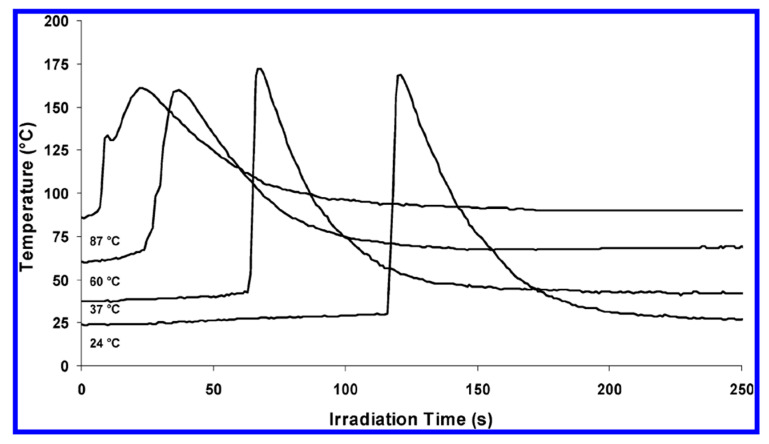
Effect of temperature on the induction time of the cationic photopolymerization of benzyl glycidyl ether. Reprinted (adapted) with permission from Umut Bulut and James V. Crivello, Investigation of the Reactivity of Epoxide Monomers in Photoinitiated Cationic Polymerization, Macromolecules, 2005, 38, 3584–3595. Copyright (2005) American Chemical Society.

In 2005, Bulut and Crivello [35] conducted a detailed study on the structure-reactivities of different epoxy monomers used in cationic photopolymerization. They also studied three different approaches for accelerating photopolymerization namely: (i) photopolymerization at elevated temperatures, (ii) copolymerization with reactive monomers, and (iii) the addition of free radical photoinitiators. First, they showed that the extended induction period in cationic photopolymerization depends on the formation of a hydronium ion crown ether species between epoxy monomer and acid proton. In the case of alkyl glycidyl ether, the hydrogen bond established between acid proton and crown ether is highly stable and prevents ring opening. However, with the increase in temperature, this induction period is greatly reduced and photopolymerization proceeds rapidly, as shown in Figure 8.

Secondly, addition of reactive monomers as copolymerization species increased the reactivity of the epoxy monomer during photopolymerization. The authors reported positive effect of trimethylolpropane trivinyl ether, 1,4-cyclohexanedimethanol divinyl ether, and diethylene glycol divinyl ether and showed by the example of NPGE and CHO that a high difference in reactivities of monomers will accelerate photopolymerization. Addition of a free radical photoinitiator (Irgacure 651) reduced the induction period during photopolymerization of NPGE and also proved to be an effective accelerator for various mono- and multifunctional glycidyl ethers [35]. Similar studies were conducted with an oxetane monomer (bis{[(1-ethyl(3-oxetanyl)]-methyl} ether) where the effect of temperature had a similar profile as in Figure 8 for benzyl glycidyl ether [120].

In the same year, Falk et al. [121] published a study on the UV-activated thermal-induced frontal polymerization for various oxirane and oxetane monomers. The group reported frontal behaviour of the epoxy monomers given in Table 1, along with their irradiation time, maximum front temperature after thermal initiation, intensity and front velocity. The formation of secondary oxonium ion species from alkyl glycidyl ether is able to portray a frontal behaviour. If sufficient energy is available in the form of heat to push secondary oxonium or tertiary oxonium ions to propagating oxonium ions in ring opening polymerization, then a frontal behaviour is observed [121]. Additionally, a similar explanation about the formation of a stabilized ether oxygen and proton bond was given, similar to the results reported by Crivello et al. [33]. Various mono- and polyfunctional alkyl glycidyl ether monomers were investigated for their reactivity in photoactivated cationic frontal polymerization by Crivello [122]. Auto-acceleration of the propagating front was achieved via thermal triggering, and it was reported that monomers exhibit frontal behaviour depending on the stability of the secondary oxonium ion intermediates through multiple hydrogen-bonding effects to the ether groups present in the molecule.

Benzyl glycidyl ether (BGE) forms a relatively less stable intermediate secondary oxonium ion in which the hydrogen ion has a bidentate coordination with oxygen in comparison to 3-cyclohexene-1,1-dimethanol diglycidyl ether (CHDMDGE) that forms tetradentate coordination. For this reason, CHDMDGE exhibits a longer induction period than BGE in the cationic photopolymerization. In addition, several epoxy monomers exhibited frontal behaviour due to their relatively stable secondary oxonium ion species in coordination with oxygen atoms. One of the epoxies is triethylene glycol diglycidyl ether (TEGDGE), which exhibited a hexadentate proton coordination and thus had a longer induction time of 200 s for UV irradiation, after which the frontal polymerization proceeded with very high temperatures. Similar longer induction periods and high photopolymerization temperatures were observed for pentaerythritol tetraglycidyl ether (PETGE), trimethylolpropane triglycidyl ether (TMPTGE), trimethylolethane triglycidyl ether (TMETGE) and glycerol triglycidyl ether (GTGE) arranged in decreasing photopolymerization temperature. Several di and tetrafunctional alkyl glycidyl ethers exhibited frontal behaviour upon thermal heating. In particular, TEGDGE and PETGE required temperatures of 69 °C and 78 °C, respectively, for thermally triggering a rapid and spontaneous frontal polymerization [122].

**Figure 9:**
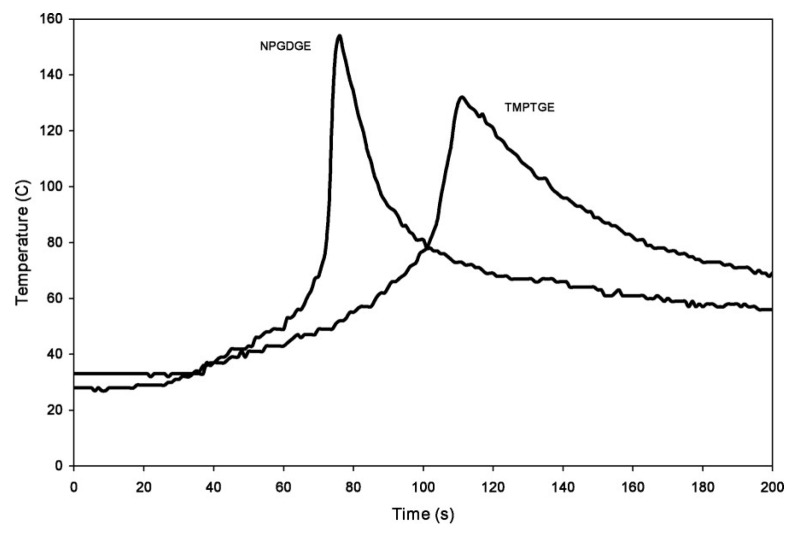
Results of the photopolymerization followed by thermal triggering for NPGDGE and TMPTGE. Extracted from James V. Crivello, Cationic photopolymerization of alkyl glycidyl ethers, Journal of Polymer Science Part A: Polymer Chemistry, 2006, 44, 3036–3052, Copyright 2006 Wiley Periodicals Inc., with permission from John Wiley and Sons.

**Figure 10:**
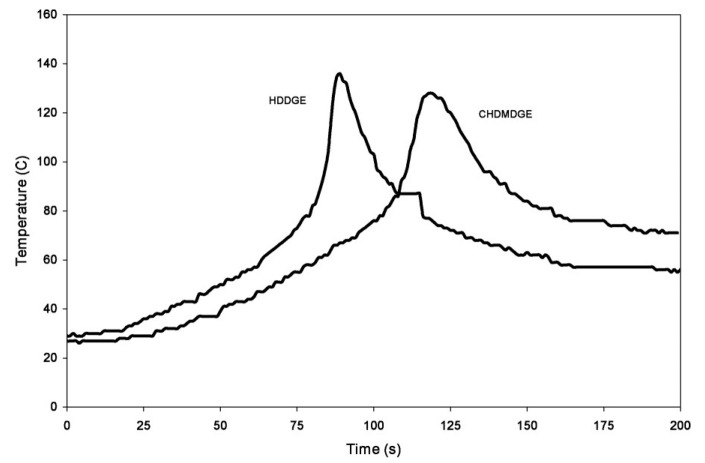
Results of photopolymerization followed by thermal triggering for HDDGE and CHDMDGE. Extracted from James V. Crivello, Cationic photopolymerization of alkyl glycidyl ethers, Journal of Polymer Science Part A: Polymer Chemistry, 2006, 44, 3036–3052, Copyright 2006 Wiley Periodicals Inc., with permission from John Wiley and Sons.

From another study done by Crivello [77], on cationic photopolymerization of alkyl glycidyl ethers, a higher cross-link density with lower front velocity was achieved with trimethylolpropane triglycidyl ether as compared to ethylene glycol diglycidyl ether. The formation of highly cross-linked structures with trimethylolpropane glycidyl ether has been attributed to a lower front velocity. Crivello also performed photopolymerization frontal experiments to confirm the effect of hydronium ion species formation on the stability of various epoxy monomers in frontal polymerization. The results shown in Figure 9 and Figure 10 clearly indicated the stability of the hydrogen-ether bond given by difference in the temperature for auto-acceleration (TMPTGE>NPGDGE in Figure 9 and 1,4-cyclohexanedimethanol diglycidyl ether CHDMDGE> 1,6-hexanediol diglycidyl ether, HDDGE, in Figure 10). It was also shown by the example of neopentylglycol diglycidyl ether, that elevated temperatures can destabilize the secondary oxonium ion species and reduce induction periods [77]. In a study conducted by Bulut and Crivello [123], it was found that benzyl sulphide is an inhibiting species in the cationic photopolymerization of BADGE and other aryl diglycidyl ethers that can inhibit polymerization of these epoxies below the gel point.

**Figure 11:**
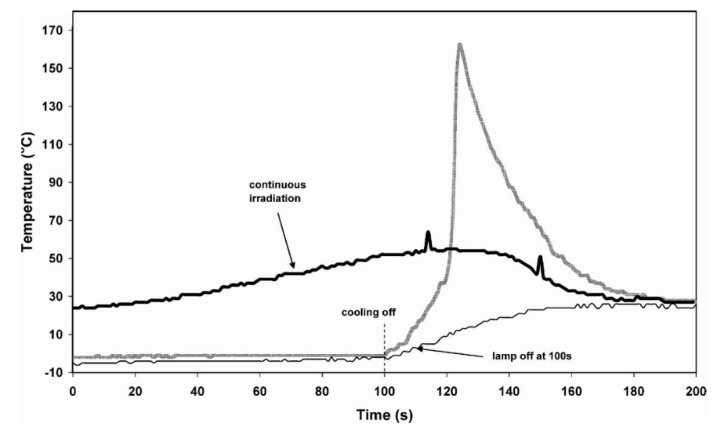
Temperature vs. time plot for frontal polymerization of glycidyl phenyl ether upon discontinuation of UV irradiation and subsequent heating after 100 s. Extracted from Effect of Temperature on the Cationic Photopolymerization of Epoxides, James V. Crivello, Journal of Macromolecular Science, Part A: Pure and Applied Chemistry, 2008, 45, 591–598, Version of record first published: 19 Jun 2008, reprinted by permission of Taylor & Francis Ltd., http://www.tandfonline.com.

In 2008, Crivello [124] published work on the effect of temperature in the cationic frontal polymerization induced by UV light on various epoxide monomers. Among these epoxide monomers, *n*-butyl glycidyl ether was UV irradiated for 200 s followed by cooling to −3 °C, and resultantly, there was no exothermic response in the temperature profile. However, a frontal polymerization was observed when the liquid film was briefly heated. The author attributed this phenomenon to the formation of a stable bidentate coordination that involves the formation of a cyclic oxonium ion, a pseudo five-membered ring by coordination with a proton. Similarly, for phenyl glycidyl ether, frontal polymerization was observed when a liquid film of the sample was heated after discontinuing UV irradiation in 100 s (Figure 11). The slow reactivity of *n*-butyl glycidyl ether as compared to phenyl glycidyl ether was attributed to the weaker bidentate coordination of protons formed by phenyl glycidyl ether with the acid proton. These results showed that continuous UV irradiation of epoxy monomers can be replaced with auto-accelerated frontal polymerization if sufficient energy is available to induce ring opening polymerization of the epoxides [124].

In 2015, Bomze et al. [38] investigated various epoxy monomers that may give a successful frontal polymerization (RICFP). Their studies showed that NPDGE, HDDGE, CHDGE and cyclo-aliphatic epoxy (CE) generated successful fronts with appreciable bubble formation, whereas combination of BADGE with tetraphenyl ethandiol and *p*-octyloxyphenyl phenyliodonium hexafluoroantimonate gave a successful front with a sluggish reactivity (2.7 cm/min) and the largest induction time among other epoxies. NPDGE possessed a high reactivity among other epoxy monomers, and this explains why it has the lowest induction period followed by HDDGE, CHDGE and CE. Front velocities for CHDGE and HDDGE were among the highest reported with 37.9 and 28.6 cm/min, respectively [38]. Bomze et al. [39] also investigated RICFP of the same formulation using BADGE to determine thermomechanical and intrinsic properties of the cured system. Their results showed that BADGE formulations consisting of TPED and iodonium salt photoinitiator can be stored at 50 °C protected from light for a period of 28 days without significant change in the viscosity and front velocity and maximum front temperatures. In comparison to thermally cured BADGE, RICFP of BADGE gave a very high T_g_ of 168 °C, tensile strength of 64 MPa and impact resistance of 14.9 kJ/m^2^ [39]. 

**Figure 12:**
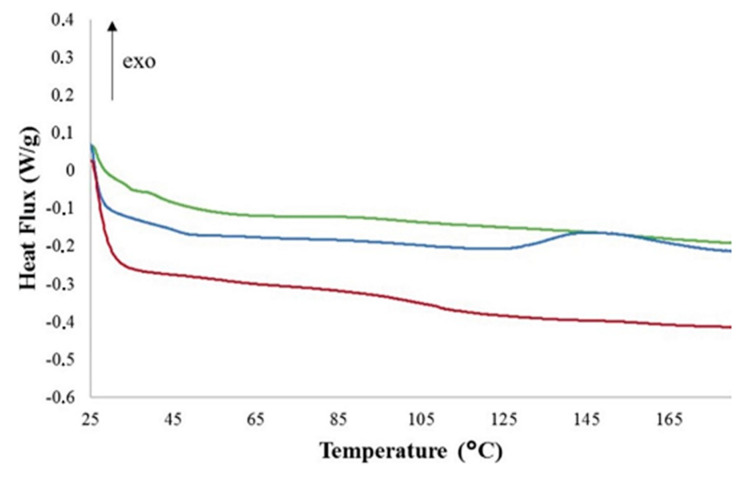
DSC plot of a formulation of BADGE with 0.5:1.5 (*w/w*) photo and thermal initiator indicating a small flex point at 150 °C (blue line) whereas 1.5:1.5 (green line) and 2:1 (*w/w*) (red line). Extracted from Marco Sangermano, Ippazio Antonazzo, Lorenzo Sisca and Massimiliana Carello, Photoinduced cationic frontal polymerization of epoxy–carbon fibre composites, Polymer International, 2019, 68, 1662–1665, Copyright 2019 Society of Chemical Industry, with permission from John Wiley and Sons.

**Figure 13:**
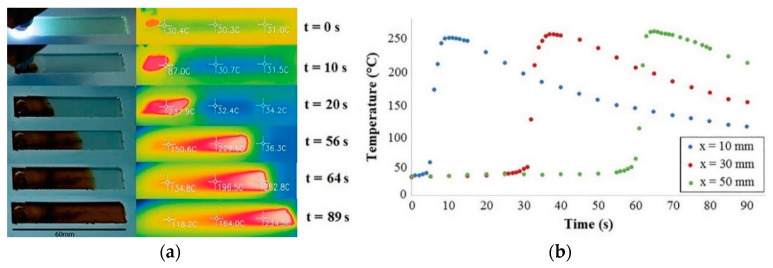
(**a**) Thermal images obtained during frontal polymerization of Ampreg 26 resin showing progressive temperature increase in the front temperature. (**b**) Temperature-time evolution of the propagating front at different distances from the irradiation point on the mould. Extracted from Marco Sangermano, Ippazio Antonazzo, Lorenzo Sisca and Massimiliana Carello, Photoinduced cationic frontal polymerization of epoxy–carbon fibre composites, Polymer International, 2019, 68, 1662–1665, Copyright 2019 Society of Chemical Industry, with permission from John Wiley and Sons.

Sangermano et al. [125] observed photoinduced cationic frontal polymerization for Ampreg 26 epoxy resin using iodonium salt photoinitiator and TPED as thermal initiator.They obtained very high curing degrees (indicated by a little or no exothermic peak in Figure 12) for the cationic polymerization of this particular epoxy resin. The frontal polymerization was monitored using a thermal camera and the temperature vs. time evolution was reported at different positions on the mould (Figure 13). The cationic polymerization of the epoxy resin proceeded with a maximum temperature of 263 °C and a frontal velocity of 4 cm/min [125].

### Effect of Diluents in UV-Induced Cationic Frontal Polymerization of Epoxide Monomers

**Figure 14:**
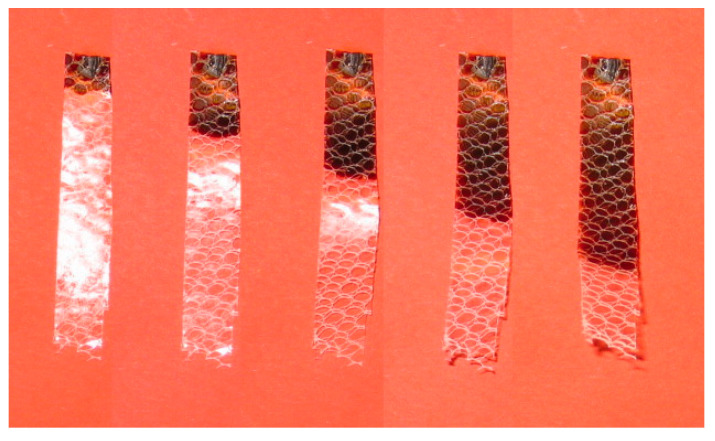
Photo-induced cationic frontal polymerization of a mixture of 5:1 TMPTGE and HDODA propagating in 10 s interval in the presence of 2-phenyl-2,2-dimethoxy-acetophenone (DMPA). Extracted from James V. Crivello, Hybrid free radical/cationic frontal photopolymerizations, Journal of Polymer Science Part A: Polymer Chemistry, 2007, 45, 4331–4340, Copyright 2007 Wiley Periodicals Inc., with permission from John Wiley and Sons.

In 2007, Crivello [126] prepared a hybrid photopolymer system consisting of a free radically polymerizable multifunctional acrylate monomer and a cationically polymerizable epoxide or oxetane monomer. Irradiation of these compounds results in the formation of a free-standing polyacrylate network film containing quiescent oxonium ions along with the unreacted cyclic ether monomer via free radical polymerization. At the same time, frontal polymerization was observed after application of a point source of heat to the film, initiating a cationic ring-opening frontal polymerization propagating to all portions of the irradiated sample (Figure 14). A 10:1 mixture of trimethylolpropane triglycidyl ether (TMPTGE) and trimethylolpropane triacrylate (TMPTA) gave a slow frontal polymerization velocity above 50 °C with a highly cross-linked epoxy network, and it was deduced that frontal polymerization requires a low concentration of acrylate monomer.

**Figure 15:**
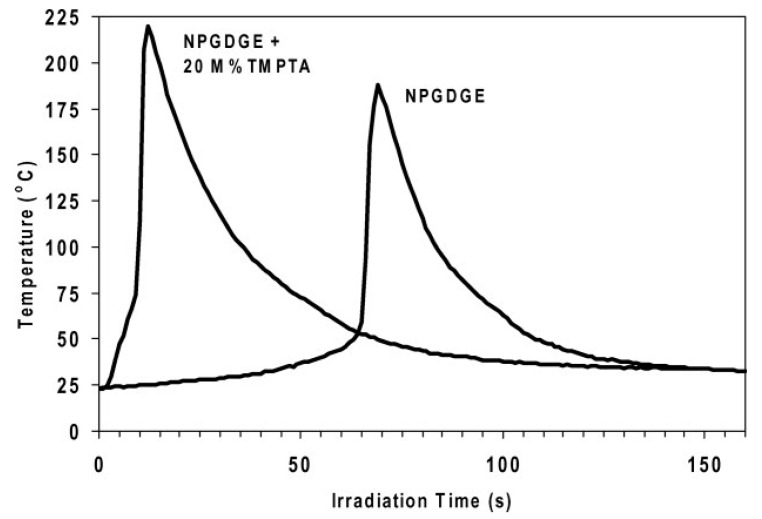
Comparison of photopolymerization of NPGDGE and multifunctional acrylate TMPTA in the presence of 2-phenyl-2,2-dimethoxy-acetophenone (DMPA) against pure NPGDGE. Extracted from James V. Crivello, Synergistic effects in hybrid free radical/cationic photopolymerizations, Journal of Polymer Science Part A: Polymer Chemistry, 2007, 45, 3759–3769, Copyright 2007 Wiley Periodicals Inc., with permission from John Wiley and Sons.

**Figure 16:**
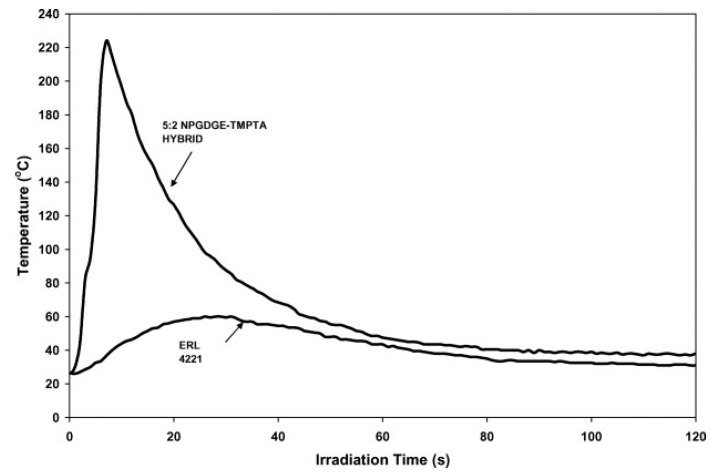
Comparison of photopolymerization of NPGDGE and multifunctional acrylate TMPTA in the presence of 2-phenyl-2,2-dimethoxy-acetophenone (DMPA) against pure ERL 4221-E. Extracted from James V. Crivello, Synergistic effects in hybrid free radical/cationic photopolymerizations, Journal of Polymer Science Part A: Polymer Chemistry, 2007, 45, 3759–3769, Copyright 2007 Wiley Periodicals Inc., with permission from John Wiley and Sons.

In contrast, 3,3-disubstituted oxetane monomers undergo rapid photoactivated cationic frontal polymerizations because of their lower inherent ring strain than epoxides and the higher basicity of the oxetane oxygen that results in the formation of stabilized tertiary oxonium ion intermediates. In a similar study, Crivello demonstrated that the addition of a multifunctional acrylate monomer in a multifunctional alkyl glycidyl ether or oxetane can accelerate the cationic photopolymerization in the presence of a free radical photoinitiator. Figure 15 and Figure 16 show a marked accelerating effect of TMPTA on the cationic polymerization of NPGDGE and ERL 4221-E [127,128].

In 2012, Ryu et al. [129] studied cationic frontal polymerization of various epoxy monomers induced by UV irradiation and followed by thermal triggering. Their approach involved a generation of supramolecular proton complexes by reversibly sequestering Brønsted acids in the presence of a cationic photo- and redox initiator system that could result in poor reactivity for the initiation of polymerization at room temperature. Frontal polymerization was observed for triethylene glycol diglycidyl ether upon contact with a hot soldering iron at a temperature of 50–70 °C after UV irradiation of the sample was discontinued. They also showed that even in the presence of a redox initiator and metal catalyst, the cationic polymerization of dipropylene glycol diglycidyl ether is accelerated upon briefly heating the solution. In addition, they also investigated the effect of addition of a crown ether that forms a hydronium ion complex with Brømsted acids. It was found that in the presence of a crown ether, the formation of a hydronium ion species entangled in a crown ether reduces the reactivity of a cyclohexene oxide monomer and an iodonium salt photoinitiator by increasing the induction time [129].

**Figure 17:**
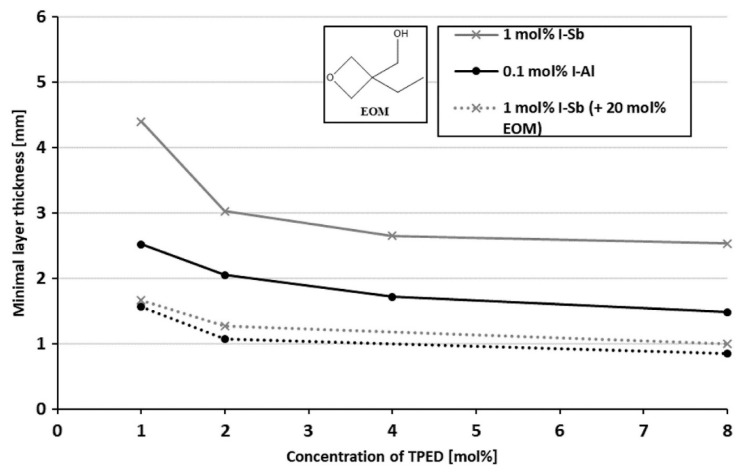
Increase in the reactivity shown by decrease in the minimal layer thickness of the cured part of a BADGE formulation consisting of 20 mol % EOM as the reactive diluent. The black dotted curve in the figure corresponds to 0.1 mol % I-Al + 20 mol % EOM. Extracted from Patrick Knaack, Nicolas Klikovits, Anh Dung Tran, Daniel Bomze, Robert Liska, Radical-induced cationic frontal polymerization in thin layers, Journal of Polymer Science Part A: Polymer Chemistry, 2019, 57, 1155–1159, Copyright 2019 The Authors. *Journal of Polymer Science Part A: Polymer Chemistry* Published by Wiley Periodicals, Inc., with permission from John Wiley and Sons.

**Figure 18:**
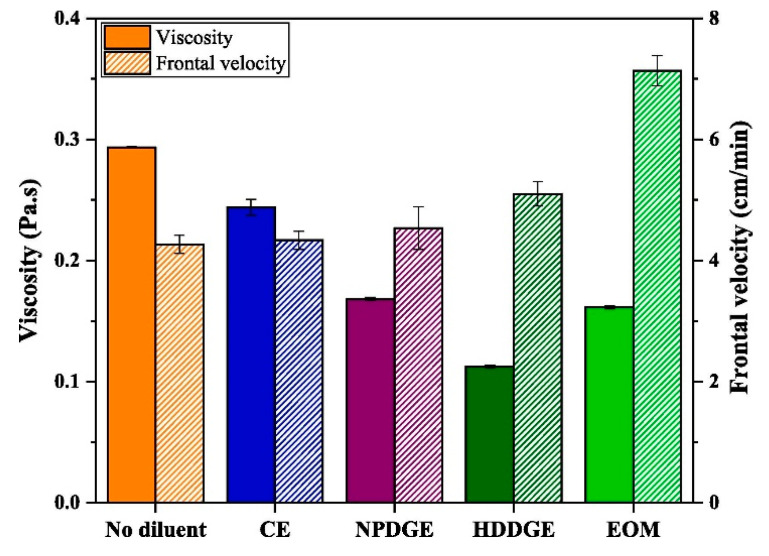
Comparison of viscosity and front velocity of several epoxy monomers in the RICFP of BADGE. Reprinted from Composites Part A, 132, Anh Dung Tran, Thomas Koch, Patrick Knaack, Robert Liska, Radical-induced cationic frontal polymerization for preparation of epoxy composites, 105855, Copyright 2020 with permission from Elsevier.

The effect of various reactive diluents on the RICFP of a formulation of BADGE, iodonium salt photoinitiator and TPED was investigated by Knaack et al. [40]. They found that (3-ethyloxetan-3-yl) methanol (EOM) gave the lowest minimal layer thickness on a PTFE mould (Figure 17). Other reactive diluents also gave lower minimal layer thickness in comparison to the reactivity of BADGE in the order of NPDGE>CHDGE>CE [40]. In a recent study by Tran et al. [41], several epoxy monomers were investigated for their effect on the RICFP of BADGE. They used 20 mol % of each of these epoxies as diluents along with 0.1 mol % of the novel I-Al photoinitiator. Among them, oxetane monomer (EOM) gave the highest reduction in viscosity and therefore the highest frontal velocity in the cationic frontal polymerization of BADGE (Figure 18). From their study, it was found that HDDGE and then EOM gave the highest heat of polymerization followed by NPDGE and CE in comparison to pure BADGE. From the storage and loss modulus curves and IR spectroscopy data obtained for each of the diluent, it was seen that a high degree of cross-linking is achieved with monomer conversion of more than 95% [41].

## 6. Effect of Fillers in Epoxy Composites

The fabrication of an epoxy composite cured via UV light was first demonstrated on glass fibre-reinforced epoxidized vegetable oils by Crivello et al. [130]. However, these composites were not frontally polymerized, and instead were irradiated for a prolonged exposure time (25 min) until the epoxy cured completely. Using a frontal polymerization technique, Bomze et al. [39] investigated the effect of mica powder on the polymerization heat consumption and light shielding effect in the RICFP of a BADGE formulation. Their studies showed that up to 20 (parts per hundred rubber, phr) of mica powder can be incorporated without a change in the viscosity of the formulation. The addition of mica to a formulation of BADGE and sensitizer did not affect the light sensitivity of the formulation and, only a slight reduction in the front velocity was observed with increasing the mica concentration from 5 to 20 phr [39]. Similar results were achieved by Knaack et al. [40], with frontal polymerization experiments on a BADGE formulation containing zirconia filler. The frontal velocity decreased from 9 cm/min to 2.8 cm/min with increasing zirconia content from 0 to 80 phr [40]. Using the same formulation of BADGE and photo and thermal radical initiator, Klikovits et al. [131], studied the effect of silica nanoparticles on frontal polymerization and mechanical properties of a BADGE-silica nanocomposite. Their studies showed that silica concentration of up to 3 phr can give a reproducible front, whereas 4 and 5 phr required a high-UV-intensity floodlight due to limited light penetration, in order to propagate the front. A decrease in the front velocity and front starting time was observed for silica concentrations up to 3 phr attributed to the low thermal conductivity and insulating properties of silica. The results from dynamic mechanical analysis showed a slight variation in the glass transition temperatures of the UV-cured epoxy nanocomposite between 132 and 141 °C [131].

**Figure 19:**
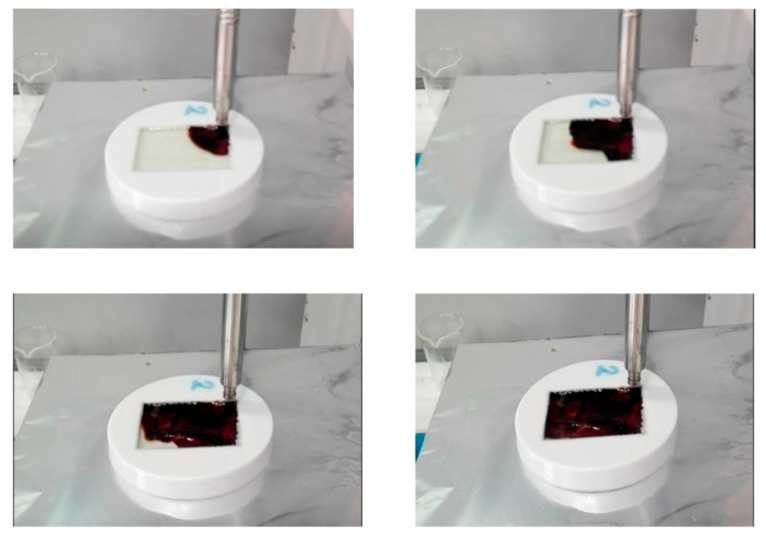
Progress of a radical-induced cationic frontal polymerization of a glass fibre-reinforced epoxy composite after 10 s of UV irradiation. Extracted from Composites Part B: Engineering, 143, M. Sangermano, A. D’Anna, C. Marro, N. Klikovits, R. Liska, UV-activated frontal polymerization of glass fibre-reinforced epoxy composites, 168–171, Copyright (2018) with permission from Elsevier.

In 2018, Sangermano et al. [42] demonstrated the possibility to UV cure a glass fibre-reinforced epoxy composite consisting of two glass-fibre layers deposited at 0° and 90°. The weight ratio for thermal to photoinitiator was set to 1:2, while the epoxy formulation consisted of a blend of bisphenol A, bisphenol F and 1,6-hexanedioldiglycidylether, which was cured within 10 s of UV exposure (Figure 19). The glass transition temperature was slightly higher (105 °C) than the thermally cured epoxy (at 95 °C). Their results also demonstrated the good applicability of UV-cured epoxy composites in comparison to thermally cured epoxy, since both types of curing methods had a slight variation in the tensile strength of the cured composite (367 MPa for UV-cured and 345 MPa for thermally cured epoxy) [42].

In another similar study, Sangermano et al. [125] prepared four piles of a 2 × 2 twill carbon fibre-reinforced epoxy composite cured via UV light and compared its properties to the corresponding thermally cured carbon-reinforced epoxy. Due to a high thermal conductivity of the carbon fibres, the front temperature and velocity was slightly higher for carbon fibre-reinforced epoxy in comparison to UV-cured epoxy without carbon fibre. In particular, the glass transition temperature of UV-cured carbon fibre-reinforced epoxy was higher than the thermally cured counterpart, which indicates a more rigid cross-linked structure. Moreover, the UV-cured epoxy composite had a tensile strength of 447 MPa in comparison to thermally cured with 715 MPa [125].

**Figure 20:**
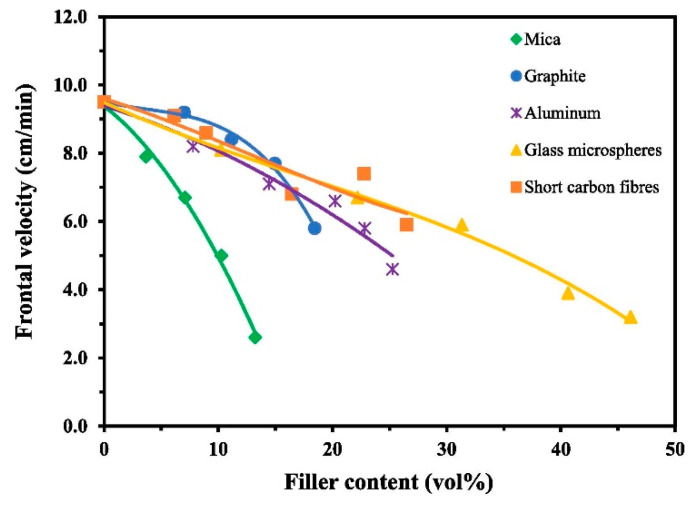
Effect of different fillers on the front velocity of a UV-cured BADGE formulation containing 1 mol % of thermal initiator TPED, 0.1 mol % photoinitiator I-Al, and 40 mol % of the diluent EOM. Reprinted from Composites Part A, 132, Anh Dung Tran, Thomas Koch, Patrick Knaack, Robert Liska, Radical-induced cationic frontal polymerization for preparation of epoxy composites, 105855, Copyright 2020 with permission from Elsevier.

**Figure 21:**
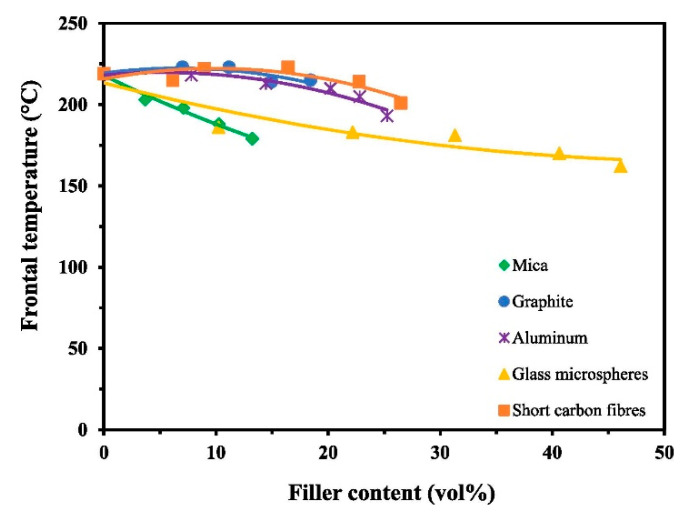
Effect of different fillers on the frontal temperature of a UV-cured BADGE formulation containing 1 mol % of thermal initiator TPED, 0.1 mol % photoinitiator I-Al, and 40 mol % of the diluent EOM. Reprinted from Composites Part A, 132, Anh Dung Tran, Thomas Koch, Patrick Knaack, Robert Liska, Radical-induced cationic frontal polymerization for preparation of epoxy composites, 105855, Copyright 2020 with permission from Elsevier.

Tran et al. [41] fabricated several filler composites using BADGE, 1 mol % of thermal initiator TPED, 0.1 mol % photoinitiator I-Al, and 40 mol % of the diluent EOM. As shown in Figure 20 and Figure 21, a general decrease in the front velocity and frontal temperature was observed for all fillers attributed to an increased heat uptake of the fillers. The authors reported that due to the high reactivity of EOM, it was possible to increase the mica content in the formulation to up to 13.2 vol%. From Figure 21, it was reported that due to high thermal conductivity of graphite, aluminium and short carbon fibre, the frontal temperature remained high at 220 °C, which sustained the front until complete curing was achieved. In addition, these fillers also gave a low void content of 2% in comparison to mica having 15% void content, which was attributed to its intrinsically high porosity. Therefore, a higher glass transition temperature was observed for composites with graphite, aluminium and short carbon fibre in comparison to mica, which gave a lower glass transition temperature than its neat formulation [41].

**Figure 22:**
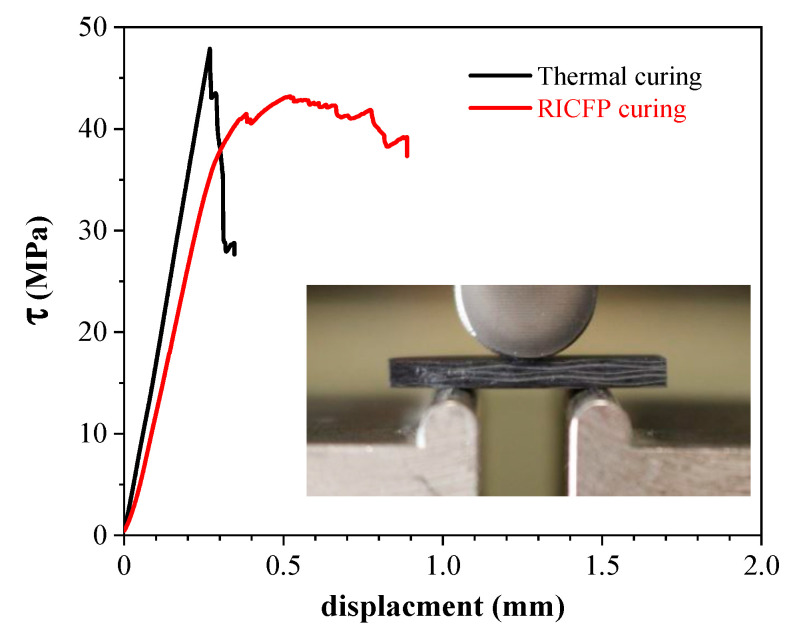
Comparison of interlaminar shear strength of thermally cured and UV-cured epoxy carbon fibre-reinforced composites. Reprinted from Composites Part A, 132, Anh Dung Tran, Thomas Koch, Patrick Knaack, Robert Liska, Radical-induced cationic frontal polymerization for preparation of epoxy composites, 105855, Copyright 2020 with permission from Elsevier.

The group also investigated the frontal and thermomechanical properties of a woven carbon fibre epoxy composite cured via UV light and compared it with its corresponding anhydride cured epoxy networks. The RICFP of the BADGE formulation gave slightly higher epoxy conversions in comparison to thermally cured specimen based on DSC and FTIR results. Though there was no significant difference between the tensile properties of UV-cured and thermally cured specimens, interlaminar shear strength values were slightly different. The failure mode of the UV-cured specimen shows multi-shear failure, with single shear being the main failure mode in the thermally cured ones (Figure 22) [41].

## 7. Conclusions and Future Perspectives

With this review, we provide a useful toolbox for the UV-triggered cationic frontal polymerization of various epoxide monomers that employ an iodonium salt photoinitiator and a thermal radical initiator. Key aspects influencing the polymerization kinetics and related thermo-mechanical properties are summarized in a comprehensive way. In particular, an overview of the chemistry of selected iodonium salts and thermal initiators is given, while studies on the cationic photopolymerization of various epoxide monomers are discussed and arranged chronologically. In addition, the use of alternative light sources for initiating the cationic polymerization is highlighted together with the effect of sensitizers on cure kinetics and front parameters. The structure–reactivity relationship and effect of reactive diluents on the polymerization of various epoxide monomers is detailed. In the last section, the transfer of the UV-triggered cationic frontal polymerization from neat resins to polymer-based composites is discussed. Particular focus is on the effect of various fillers and fibres on the radical-induced cationic frontal polymerization of epoxy-based resin formulations.

Cationic frontal polymerization of epoxide monomers has opened up a new path towards a rapid, low cost and energy-independent curing of epoxy-based resins. Recent studies by Knaack et al. [40], predict the potential applications of cationic frontal polymerization, demonstrating excellent performance of frontally cured epoxy resins in chemical anchor bolts and underwater crack filling of various materials. However, the implementation of this curing technique in various industrial applications is yet to be achieved. Various potential applications of frontal polymerization were also compiled by Pojman [8] in his review, where applications such as hydrogels, consolidation of stone, autoclave-less curing of large composites and cure-on-demand repair and adhesives were discussed. Robertson et al. [132] demonstrated a thermally initiated frontal ring opening metathesis polymerization (FROMP) of dicyclopentadiene, which was successfully used to fabricate a 900 cm^2^ corrugated carbon fibre-reinforced polymer composite (FRPC) panel in only 5 min and by spending 750 J of energy. From the results of a study on the energy consumed versus time required to cure composites, the group calculated that the curing of a section of the Boeing 787 fuselage by FROMP reduces the energy consumption by ten orders of magnitude compared with autoclave processing technique [132].

In the production of composite materials, the product cycle time, total energy consumption, fibre–matrix adhesion and the quality of the cured composite are crucial parameters that are presently a subject of improvement via an intensive researching. Particularly for thermosetting resins such as epoxide monomers, maximum monomer conversion or high cross-link density and uniform distribution within the fibre/filler can be improved by employing frontal polymerization. This technique does not only improve the final product quality but also cuts down a lot in terms of input energy requirements for thermal curing and subsequent post curing, as well as eliminating the usage of toxic solvents for dissolution of the curing agents. Furthermore, complex part geometries that are either not fully accessible during a conventional curing process can now be easily accessed via frontal polymerization at ambient processing temperature and without the necessity of tools for curing under high-pressure.

The kinetics of frontal polymerization allows the use of various other monomers that are particularly used to improve the mechanical properties of neat epoxy resins. This means that any functionalized monomer that do not act as radical scavengers during the initial photo cleavage of the iodonium salt and also has appreciable polarity to solubilize with epoxy monomers can be used to prepare epoxy blends of desired thermomechanical properties. Epoxidized elastomers are an interesting class of monomers that can be employed also in the fabrication of high tensile and impact strength composites, which were firstly reported by Crivello and Yang in 1994 [133]. Due to the development of various visible light absorbing photoinitiators, the epoxy photopolymerization technology also has a potential to be expanded towards green synthesis pathways using sunlight as light source. Combined with epoxidized vegetable oils, which were firstly studied by Crivello et al. [106,130], green composites can be literally fabricated using natural fibres and sunlight only, which may find applications in sailing boats and roofing surfaces for homes. The on-site damage repair of epoxy composites as well as epoxy-based electrical insulating materials using frontal polymerization is also a promising alternative to conventional repairing processes providing rapid, low-cost and uniform curing of the damaged materials. With the photoinduced frontal polymerization technique, defects within the composite part can be rapidly fixed by injecting a small volume of the epoxy-initiator mixed solution filling the cracks and then allowing it to cure frontally with a local UV irradiation.

Frontal polymerization was also acknowledged by Pojman et al. [134] as a potential technique for in-space repair and fabrication. In fact, studies on the possibility of employing frontal polymerization for aeronautics and space materials were also reported with positive results from orbital experiments and terrestrial simulations in the past few decades [135,136]. Cationic photopolymerization has been demonstrated in the past among materials used in various space missions such as the STS-46 by NASA (National Aeronautics and Space Administration), where epoxy-functionalized siloxanes were employed as a protective coating against atomic oxygen [137]. These materials were successfully used to fabricate composites and transparent coatings for the space shuttle providing excellent coating resistance to outer space debris and micrometeoroids. We anticipate that with the currently increasing space missions organized by various aerospace institutes around the globe, the photoinduced frontal polymerization technology will play an active role in providing an economic and engineered performance for the various epoxy/fibre composites used.

With recent successful studies focused on the thermomechanical properties of fibre/filler-reinforced epoxy composites [39,40,41,42,125], we predict that radical-induced cationic frontal polymerization will prove to be a promising curing technology providing energy efficient process, reduced cycle time and excellent product performance.

## Figures and Tables

**Table 1:** Different epoxy monomers displaying frontal behaviour during cationic photopolymerization coupled with thermal initiation. Redrawn from Benjamin Falk, Michael R. Zonca, Jr., James V. Crivello, Photoactivated Cationic Frontal Polymerization, Macromolecular Symposia, 2005, 226, 97–107, Copyright 2005 International Union of Pure and Applied Chemistry, with permission from John Wiley and Sons.

Monomer	Irrad. Time (min)	Intensity (mJ/cm^2^min)	T_f_ (^o^C)	t_f_ (s)	V_f_ (cm/min)
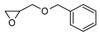	2.5	439	180	35	7.2
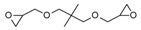	6	413	232	22	11
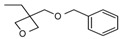	5	483	169	14	18
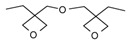	8	575	203	6	20

## References

[1] Chen S., Sui J., Chen L., Pojman J.A. (2005). Polyurethane-nanosilica hybrid nanocomposites synthesized by frontal polymerization. J. Polym. Sci. Part A Polym. Chem..

[2] Chekanov Y.A., Pojman J.A. (2000). Preparation of functionally gradient materials via frontal polymerization. J. Appl. Polym. Sci..

[3] Guo X., Wang C.-F., Fang Y., Chen L., Chen S. (2011). Fast synthesis of versatile nanocrystal-embedded hydrogels toward the sensing of heavy metal ions and organoamines. J. Mater. Chem..

[4] Yan Q.-Z., Zhang W., Lu G.-D., Su X.-T., Ge C.-C. (2006). Frontal Polymerization Synthesis of Starch-Grafted Hydrogels: Effect of Temperature and Tube Size on Propagating Front and Properties of Hydrogels. Chem. A Eur. J..

[5] Sanna R., Alzari V., Nuvoli D., Scognamillo S., Marceddu S., Mariani A. (2012). Polymer hydrogels of 2-hydroxyethyl acrylate and acrylic acid obtained by frontal polymerization. J. Polym. Sci. Part A Polym. Chem..

[6] Nagy I.P., Sike L., Pojman J.A. (1995). Thermochromic Composite Prepared via a Propagating Polymerization Front. J. Am. Chem. Soc..

[7] White S.R., Kim C. (1993). A Simultaneous Lay-Up and in situ Cure Process for Thick Composites. J. Reinf. Plast. Compos..

[8] Pojman J.A. (2012). Frontal Polymerization. Polymer Science: A Comprehensive Reference.

[9] Chechilo N.M., Khvilivitskii R.J., Enikolopyan N.S. (1972). On the Phenomenon of Polymerization Reaction Spreading. Dokl. Akad. Nauk SSSR.

[10] Davtyan D.S., Tonoyan A.O., Davtyan S.P., Savchenko V.I. (1999). Geometric shape and stability of frontal regimes during radical polymerization of MMA in cylindrical flow reactor. Polym. Sci. Ser. A.

[11] Davtyan D.S., Tonoyan A.O., Radugina A.A., Davtyan S.P., Savchenko V.I., Abrosimov A.F. (1999). Control of conversion and molecular masses during the frontal polymerization of MMA in a cylindrical flow reactor. Polym. Sci. Ser. A.

[12] Davtyan D.S., Tonoyan A.O., Radugina A.A., Davtyan S.P., Savchenko V.I., Abrosimov A.F. (1999). Frontal radical polymerization of methyl methacrylate in a cylindrical flow reactor. Polym. Sci. Ser. A.

[13] Decker C. (1996). Photoinitiated crosslinking polymerisation. Prog. Polym. Sci..

[14] Peiffer R.W., Scranton A.B., Bowman C.N., Peiffer R.W. (1997). Applications of photopolymer technology. Photopolymerization: Fundamentals and Applications.

[15] Fouassier J.P., Rabek J.F. (1993). Radiation Curing in Polymer Science and Technology.

[16] Pappas S.P. (1992). Radiation Curing. Science and Technology.

[17] Green W.A. (2010). Industrial Photoinitiators. A Technical Guide.

[18] Crivello J.V., Lam J.H.W. (1977). New photoinitiators for cationic polymerization. J. Polym. Sci. Symp..

[19] Crivello J.V., Lam J.H.W. (1977). Diaryliodonium Salts. A New Class of Photoinitiators for Cationic Polymerization. Macromolecules.

[20] Crivello J.V., Lam J.H.W. (1979). Photoinitiated cationic polymerization with triarylsulfonium salts. J. Polym. Sci. Polym. Chem. Ed..

[21] Crivello J.V., Lam J.H.W. (1978). Dye-sensitized photoinitiated cationic polymerization. J. Polym. Sci. Polym. Chem. Ed..

[22] Crivello J.V. (2012). Photopolymerization. Polymer Science: A Comprehensive Reference.

[23] Crivello J.V. (1999). The discovery and development of onium salt cationic photoinitiators. J. Polym. Sci. Part A Polym. Chem..

[24] Sangermano M. (2012). Advances in cationic photopolymerization. Pure Appl. Chem..

[25] Sangermano M., Razza N., Crivello J.V. (2014). Cationic UV-Curing: Technology and Applications. Macromol. Mater. Eng..

[26] Kaur M., Srivastava A.K. (2002). Photopolymerization: A Review. J. Macromol. Sci. Part C Polym. Rev..

[27] Crivello J.V., Lam J.H.W., Scranton A.B., Bowman C.N., Peiffer R.W. (1997). The photoinitiated cationic polymerization of epoxy resins. Photopolymerization: Fundamentals and Applications.

[28] Zopf R.F. (1982). UV Cationic Epoxy Systems for Dielectric Applications. Radiat. Curing.

[29] Smith G.H. (1981). Photocopolymerizable Compositions Based on Epoxy and Hydroxyl-Containing Organic Materials. US Patent.

[30] Wang A.A., Knopf R.J., Osborn C.L. (1981). Cationically Polymerizable Radiation Curable Compositions. Patent.

[31] Fouassier J.-P., Lalevée J. (2012). Photoinitiators for Polymer Synthesis. Scope, Reactivity and Efficiency.

[32] Penczek S., Kubisa P., Matyjaszewski K. (1980). Cationic Ring-Opening Polymerization of Heterocyclic Monomers.

[33] Crivello J.V., Falk B., Zonca M.R. (2004). Photoinduced cationic ring-opening frontal polymerizations of oxetanes and oxiranes. J. Polym. Sci. Part A Polym. Chem..

[34] Crivello J. (2005). Investigation of the photoactivated frontal polymerization of oxetanes using optical pyrometry. Polymer.

[35] Bulut U., Crivello J.V. (2005). Investigation of the Reactivity of Epoxide Monomers in Photoinitiated Cationic Polymerization. Macromolecules.

[36] Mariani A., Bidali S., Fiori S., Sangermano M., Malucelli G., Bongiovanni R., Priola A. (2004). UV-ignited frontal polymerization of an epoxy resin. J. Polym. Sci. Part A Polym. Chem..

[37] Brandrup J., Immergut E.H. (1966). Polymer Handbook.

[38] Bomze D., Knaack P., Liska R. (2015). Successful radical induced cationic frontal polymerization of epoxy-based monomers by C–C labile compounds. Polym. Chem..

[39] Bomze D., Knaack P., Koch T., Jin H., Liska R. (2016). Radical induced cationic frontal polymerization as a versatile tool for epoxy curing and composite production. J. Polym. Sci. Part A Polym. Chem..

[40] Knaack P., Klikovits N., Tran A.D., Bomze D., Liska R. (2019). Radical induced cationic frontal polymerization in thin layers. J. Polym. Sci. Part A Polym. Chem..

[41] Tran A.D., Koch T., Knaack P., Liska R. (2020). Radical induced cationic frontal polymerization for preparation of epoxy composites. Compos. Part A Appl. Sci. Manuf..

[42] Sangermano M., D’Anna A., Marro C., Klikovits N., Liska R. (2018). UV-activated frontal polymerization of glass fibre reinforced epoxy composites. Compos. Part B Eng..

[43] Sangermano M., Roppolo I., Chiappone A. (2018). New Horizons in Cationic Photopolymerization. Polymer.

[44] Atif M., Bongiovanni R., Yang J. (2015). Cationically UV-Cured Epoxy Composites. Polym. Rev..

[45] Strohmeier V.W., Barbeau C. (1965). Polymerisation von Propylenoxid mit Mangandecacarbonyl nach UV-Bestrahlung. Die Makromol. Chem..

[46] Kaeriyama K. (1976). Photocatalyzed polymerization of epichlorohydrin. J. Polym. Sci. Polym. Chem. Ed..

[47] Licari J.J., Crepeau P.C. (1965). Electromagnetic Radiation Polymerization. US Patent.

[48] Schlesinger S.I. (1974). Photopolymerization of Epoxides. Photogr. Sci. Eng..

[49] Crivello J.V., Lee J.L. (1989). Alkoxy-substituted diaryliodonium salt cationic photoinitiators. J. Polym. Sci. Part A Polym. Chem..

[50] Crivello J.V. (2008). Design and Synthesis of Photoacid Generating Systems. J. Photopolym. Sci. Technol..

[51] Crivello J.V. (1984). Cationic polymerization–Iodonium and sulfonium salt photoinitiators. Initiators—Poly-Reactions—Optical Activity.

[52] Crivello J.V., Lam J.H.W., Volante C.N. (1977). Photoinitiated Cationic Polymerization using Diaryliodonium Salts. J. Radiat. Curing.

[53] Crivello J.V., Lam J.H.N., Moore J.E., Schroeter S.H. (1978). Triarylsulfonium Salts: A New Class of Photoinitiators for Cationic Polymerization. J. Radiat. Curing.

[54] Crivello J.V., Lee J.L. (1990). The synthesis, characterization, and photoinitiated cationic polymerization of silicon-containing epoxy resins. J. Polym. Sci. Part A Polym. Chem..

[55] Castellanos F., Fouassier J.P., Priou C., Cavezzan J. (1996). Synthesis, reactivity, and properties of new diaryliodonium salts as photoinitiators for the cationic polymerization of epoxy silicones. J. Appl. Polym. Sci..

[56] Klikovits N., Knaack P., Bomze D., Krossing I., Liska R. (2017). Novel photoacid generators for cationic photopolymerization. Polym. Chem..

[57] Wang D., Szillat F., Fouassier J.P., Lalevée J. (2019). Remarkable Versatility of Silane/Iodonium Salt as Redox Free Radical, Cationic, and Photopolymerization Initiators. Macromolecules.

[58] Yagci Y., Hepuzer Y. (1999). A Novel Visible Light Initiatiating System for Cationic Polymerization. Macromolecules.

[59] Fouassier J.P. (1995). Photoinitiator, Photopolymerization and Photocuring: Fundamentals and Applications.

[60] Pappas S.P., Gatechair L.R., Jilek J.H. (1984). Photoinitiation of cationic polymerization. III. Photosensitization of diphenyliodonium and triphenylsulfonium salts. J. Polym. Sci. Polym. Chem. Ed..

[61] Yaǧci Y., Schnabel W., Yağcı Y. (1994). Direct and sensitized photoinitiated cationic polymerization using pyridinium salts. Macromol. Symp..

[62] Yagci Y., Endo T., Améduri B. (1997). N-benzyl and N-alkoxy pyridinium salts as thermal and photochemical initiators for cationic polymerization. Polymer Synthesis: Polymer Catalysis.

[63] Bi Y., Neckers D.C. (1994). A Visible Light Initiating System for Free Radical Promoted Cationic Polymerization. Macromolecules.

[64] Chen Y., Yamamura T., Igarashi K. (2000). Photosensitization of carbazole derivatives in cationic polymerization with a novel sensitivity to near-UV light. J. Polym. Sci. Part A Polym. Chem..

[65] Hua Y., Crivello J.V. (2000). Synergistic interaction of epoxides andN-vinylcarbazole during photoinitiated cationic polymerization. J. Polym. Sci. Part A Polym. Chem..

[66] Hua Y., Crivello J.V. (2001). Development of Polymeric Photosensitizers for Photoinitiated Cationic Polymerization. Macromolecules.

[67] Crivello J.V., Sangermano M. (2001). Visible and long-wavelength photoinitiated cationic polymerization. J. Polym. Sci. Part A Polym. Chem..

[68] Hua Y., Jiang F., Crivello J.V. (2002). Photosensitized Onium-Salt-Induced Cationic Polymerization with Hydroxymethylated Polynuclear Aromatic Hydrocarbons. Chem. Mater..

[69] Gomurashvili Z., Crivello J.V. (2001). Phenothiazine photosensitizers for onium salt photoinitiated cationic polymerization. J. Polym. Sci. Part A Polym. Chem..

[70] Gomurashvili Z., Crivello J.V. (2002). Monomeric and Polymeric Phenothiazine Photosensitizers for Photoinitiated Cationic Polymerization. Macromolecules.

[71] Crivello J.V., Jiang F. (2002). Development of Pyrene Photosensitizers for Cationic Photopolymerizations. Chem. Mater..

[72] Crivello J.V., Jang M. (2003). Anthracene electron-transfer photosensitizers for onium salt induced cationic photopolymerizations. J. Photochem. Photobiol. A Chem..

[73] Falk B., Vallinas S.M., Crivello J.V. (2003). Monitoring photopolymerization reactions with optical pyrometry. J. Polym. Sci. Part A Polym. Chem..

[74] Crivello J.V., Jang M. (2005). Synthesis of Monomer and Polymer-Bound Photosensitizers. J. Macromol. Sci. Part A.

[75] Crivello J.V., Bulut U. (2005). Curcumin: A naturally occurring long-wavelength photosensitizer for diaryliodonium salts. J. Polym. Sci. Part A Polym. Chem..

[76] Crivello J.V., Bulut U. (2006). Indian Turmeric and its Use in Cationic Photopolymerizations. Macromol. Symp..

[77] Crivello J.V. (2006). Cationic photopolymerization of alkyl glycidyl ethers. J. Polym. Sci. Part A Polym. Chem..

[78] Crivello J.V. (2008). A new visible light sensitive photoinitiator system for the cationic polymerization of epoxides. J. Polym. Sci. Part A Polym. Chem..

[79] Crivello J.V. (2009). Radical-Promoted Visible Light Photoinitiated Cationic Polymerization of Epoxides. J. Macromol. Sci. Part A.

[80] Schroeder W.F., Asmussen S.V., Sangermano M., Vallo C.I. (2013). Visible light polymerization of epoxy monomers using an iodonium salt with camphorquinone/ethyl-4-dimethyl aminobenzoate. Polym. Int..

[81] Liu G., Zhu X., Xu B., Qian X., Song G.-Q., Nie J. (2013). Cationic photopolymerization of bisphenol A diglycidyl ether epoxy under 385 nm. J. Appl. Polym. Sci..

[82] Telitel S., Blanchard N., Schweizer S., Morlet-Savary F., Graff B., Fouassier J.-P., Lalevée J. (2013). BODIPY derivatives and boranil as new photoinitiating systems of cationic polymerization exhibiting a tunable absorption in the 400–600 nm spectral range. Polymer.

[83] Al Mousawi A., Garra P., Dumur F., Bui T.-T., Goubard F., Toufaily J., Hamieh T., Graff B., Gigmes D., Fouassier J.P. (2017). Novel Carbazole Skeleton-Based Photoinitiators for LED Polymerization and LED Projector 3D Printing. Molecules.

[84] Al Mousawi A., Arar A., Ibrahim-Ouali M., Duval S., Dumur F., Garra P., Toufaily J., Hamieh T., Graff B., Gigmes D. (2018). Carbazole-based compounds as photoinitiators for free radical and cationic polymerization upon near visible light illumination. Photochem. Photobiol. Sci..

[85] Al Mousawi A., Dumur F., Garra P., Toufaily J., Hamieh T., Graff B., Gigmes D., Fouassier J.P., Lalevée J. (2017). Carbazole Scaffold Based Photoinitiator/Photoredox Catalysts: Toward New High Performance Photoinitiating Systems and Application in LED Projector 3D Printing Resins. Macromolecules.

[86] Xiao P., Lalevée J., Zhao J., Stenzel M.H. (2015). N -Vinylcarbazole as Versatile Photoinaddimer of Photopolymerization under Household UV LED Bulb (392 nm). Macromol. Rapid Commun..

[87] Sangermano M., Rodríguez D., Gonzalez M.C., Laurenti E., Yagci Y. (2018). Visible Light Induced Cationic Polymerization of Epoxides by Using Multiwalled Carbon Nanotubes. Macromol. Rapid Commun..

[88] Zhang J., Dumur F., Horcajada P., Livage C., Xiao P., Fouassier J.P., Gigmes D., Lalevée J. (2016). Iron-Based Metal-Organic Frameworks (MOF) as Photocatalysts for Radical and Cationic Polymerizations under Near UV and Visible LEDs (385–405 nm). Macromol. Chem. Phys..

[89] Al Mousawi A., Lara D.M., Noirbent G., Dumur F., Toufaily J., Hamieh T., Bui T.-T., Goubard F., Graff B., Gigmes D. (2017). Carbazole Derivatives with Thermally Activated Delayed Fluorescence Property as Photoinitiators/Photoredox Catalysts for LED 3D Printing Technology. Macromolecules.

[90] Mokbel H., Anderson D., Plenderleith R., Dietlin C., Morlet-Savary F., Dumur F., Gigmes D., Fouassier J.-P., Lalevée J. (2017). Copper photoredox catalyst “G1”: A new high performance photoinitiator for near-UV and visible LEDs. Polym. Chem..

[91] Al Mousawi A., Garra P., Sallenave X., Dumur F., Toufaily J., Hamieh T., Graff B., Gigmes D., Fouassier J.P., Lalevée J. (2018). π-Conjugated Dithienophosphole Derivatives as High Performance Photoinitiators for 3D Printing Resins. Macromolecules.

[92] Zhang J., Campolo D., Dumur F., Xiao P., Fouassier J.P., Gigmes D., Lalevée J. (2016). Iron Complexes in Visible-Light-Sensitive Photoredox Catalysis: Effect of Ligands on Their Photoinitiation Efficiencies. ChemCatChem.

[93] Al Mousawi A., Dumur F., Garra P., Toufaily J., Hamieh T., Goubard F., Bui T.-T., Graff B., Gigmes D., Fouassier J.P. (2017). Azahelicenes as visible light photoinitiators for cationic and radical polymerization: Preparation of photoluminescent polymers and use in high performance LED projector 3D printing resins. J. Polym. Sci. Part A Polym. Chem..

[94] Xiao P., Zhang J., Graff B., Fouassier J.P., Lalevée J. (2017). Rubrene-Based Green-Light-Sensitive Photoinitiating Systems of Polymerization. Macromol. Chem. Phys..

[95] Zhang J., Zivic N., Dumur F., Xiao P., Graff B., Fouassier J.P., Gigmes D., Lalevée J. (2018). N-[2-(Dimethylamino)ethyl]-1,8-naphthalimide derivatives as photoinitiators under LEDs. Polym. Chem..

[96] Mokbel H., Poriel C., Rault-Berthelot J., Dumur F., Gigmes D., Toufaily J., Hamieh T., Cordella D., Detrembleur C., Fouassier J.P. (2016). A glance at violet LED sensitive photoinitiators based on the spiroxanthene scaffold. J. Appl. Polym. Sci..

[97] Mokbel H., Xiao P., Simonnet-Jégat C., Dumur F., Gigmes D., Toufaily J., Hamieh T., Fouassier J.P., Lalevée J. (2015). Iodonium-polyoxometalate and thianthrenium-polyoxometalate as new one-component UV photoinitiators for radical and cationic polymerization. J. Polym. Sci. Part A Polym. Chem..

[98] Meng X., Lu H., Li Z., Wang C., Liu R., Guan X., Yagci Y. (2019). Near-infrared light induced cationic polymerization based on upconversion and ferrocenium photochemistry. Polym. Chem..

[99] Bouzrati-Zerelli M., Guillaume N., Goubard F., Bui T.-T., Villotte S., Dietlin C., Morlet-Savary F., Gigmes D., Fouassier J.P., Dumur F. (2018). A novel class of photoinitiators with a thermally activated delayed fluorescence (TADF) property. New J. Chem..

[100] Kaya K., Seba M., Fujita T., Yamago S., Yagci Y. (2018). Visible light-induced free radical promoted cationic polymerization using organotellurium compounds. Polym. Chem..

[101] Kaya K., Kreutzer J., Yagci Y. (2019). A Charge-Transfer Complex of Thioxanthonephenacyl Sulfonium Salt as a Visible-Light Photoinitiator for Free Radical and Cationic Polymerizations. ChemPhotoChem.

[102] Wang D., Kaya K., Garra P., Fouassier J.P., Graff B., Yagci Y., Lalevée J. (2019). Sulfonium salt based charge transfer complexes as dual thermal and photochemical polymerization initiators for composites and 3D printing. Polym. Chem..

[103] Garra P., Caron A., Al Mousawi A., Graff B., Morlet-Savary F., Dietlin C., Yagci Y., Fouassier J.-P., Lalevée J. (2018). Photochemical, Thermal Free Radical, and Cationic Polymerizations Promoted by Charge Transfer Complexes: Simple Strategy for the Fabrication of Thick Composites. Macromolecules.

[104] Zhou J., Jia S., Fu W., Liu Z., Tan Z. (2016). Fast curing of thick components of epoxy via modified UV-triggered frontal polymerization propagating horizontally. Mater. Lett..

[105] Crivello J.V., Lee J.L. (1986). The synthesis and characterization of polymer-bound diaryliodonium salts and their use in photo and thermally initiated cationic polymerization. Polym. Bull..

[106] Crivello J.V., Narayan R. (1992). Epoxidized triglycerides as renewable monomers in photoinitiated cationic polymerization. Chem. Mater..

[107] Crivello J.V. (1992). The synthesis and cationic polymerization of novel epoxide monomers. Polym. Eng. Sci..

[108] Crivello J.V., Bi D. (1993). Regioselective hydrosilations. IV. The synthesis and polymerization of monomers containing epoxy and alkoxysilane groups. J. Polym. Sci. Part A Polym. Chem..

[109] Crivello J.V., Carter A.M. (1993). Photochemical synthesis and cationic photopolyermization of α,ω-diepoxyalkanes. J. Polym. Sci. Part A Polym. Chem..

[110] Crivello J.V., Bi D. (1994). The synthesis and cationic polymerization of multifunctional silicon-containing epoxy monomers and oligomers. J. Polym. Sci. Part A Polym. Chem..

[111] Crivello J.V., Kim W. (1994). Synthesis and photopolymerization of 1-propenyl glycidyl ether. J. Polym. Sci. Part A Polym. Chem..

[112] Crivello J., Bi D., Fan M. (1994). Synthesis and Polymerization of Novel Cationically Polymerizable Monomers. J. Macromol. Sci. Part A.

[113] Crivello J.V., Malik R. (1997). Synthesis and photoinitiated cationic polymerization of monomers with the silsesquioxane core. J. Polym. Sci. A Polym. Chem..

[114] Crivello J.V., Liu S.S. (1998). Synthesis and cationic photopolymerization of 1-butenyl glycidyl ether. J. Polym. Sci. Part A Polym. Chem..

[115] Crivello J.V., Varlemann U., Scranton A.B., Bowman C.N., Peiffer R.W. (1997). Structure and reactivity relationships in the photoinitiated cationic polymerization of 3,4-epoxycyclohexylmethyl-3′,4′-epoxycyclohexane carboxylate. Photopolymerization: Fundamentals and Applications.

[116] Crivello J.V., Varlemann U. (1995). The synthesis and study of the photoinitiated cationic polymerization of novel cycloaliphatic epoxides. J. Polym. Sci. Part A Polym. Chem..

[117] Crivello J.V., Varlemann U. (1995). Mechanistic study of the reactivity of 3,4-epoxycyclohexylmethyl 3′,4′-epoxycyclohexancarboxylate in photoinitiated cationic polymerizations. J. Polym. Sci. Part A Polym. Chem..

[118] Crivello J.V., Liu S. (2000). Photoinitiated cationic polymerization of epoxy alcohol monomers. J. Polym. Sci. Part A Polym. Chem..

[119] Crivello J.V., Falk B., Zonca M.R. (2004). Study of cationic ring-opening photopolymerizations using optical pyrometry. J. Appl. Polym. Sci..

[120] Bulut U., Crivello J.V. (2005). Reactivity of oxetane monomers in photoinitiated cationic polymerization. J. Polym. Sci. Part A Polym. Chem..

[121] Falk B., Zonca M.R., Crivello J.V. (2005). Photoactivated Cationic Frontal Polymerization. Macromol. Symp..

[122] Crivello J.V. (2006). Design and synthesis of multifunctional glycidyl ethers that undergo frontal polymerization. J. Polym. Sci. Part A Polym. Chem..

[123] Crivello J.V., Bulut U. (2006). Dual photo- and thermally initiated cationic polymerization of epoxy monomers. J. Polym. Sci. Part A Polym. Chem..

[124] Crivello J.V. (2008). Effect of Temperature on the Cationic Photopolymerization of Epoxides. J. Macromol. Sci. Part A.

[125] Sangermano M., Antonazzo I., Sisca L., Carello M. (2019). Photoinduced cationic frontal polymerization of epoxy–carbon fibre composites. Polym. Int..

[126] Crivello J.V. (2007). Hybrid free radical/cationic frontal photopolymerizations. J. Polym. Sci. Part A Polym. Chem..

[127] Crivello J.V. (2007). Synergistic effects in hybrid free radical/cationic photopolymerizations. J. Polym. Sci. Part A Polym. Chem..

[128] Crivello J.V. (2014). Hybrid acrylate-oxetane photopolymerizable systems. J. Polym. Sci. Part A Polym. Chem..

[129] Ryu C.Y., Spencer M.J., Crivello J.V. (2012). Involvement of Supramolecular Complexes in the Capture and Release of Protonic Acids During the Cationic Ring-Opening Polymerization of Epoxides. Macromolecules.

[130] Crivello J.V., Narayan R., Sternstein S. (1997). Fabrication and mechanical characterization of glass fiber reinforced UV-cured composites from epoxidized vegetable oils. J. Appl. Polym. Sci..

[131] Klikovits N., Liska R., D’Anna A., Sangermano M. (2017). Successful UV-Induced RICFP of Epoxy-Composites. Macromol. Chem. Phys..

[132] Robertson I.D., Yourdkhani M., Centellas P.J., Aw J.E., Ivanoff D.G., Goli E., Lloyd E.M., Dean L.M., Sottos N.R., Geubelle P. (2018). Rapid energy-efficient manufacturing of polymers and composites via frontal polymerization. Nature.

[133] Crivello J.V., Yang B. (1994). Synthesis and Photoinitiated Cationic Polymerization of Epoxidized Elastomers. J. Macromol. Sci. Part A.

[134] Pojman J.A., Aerospace Sciences Meetings (2005). 43rd AIAA Aerospace Sciences Meeting and Exhibit.

[135] Briskman V.A., Kostarev K.G., Yudina T.M., Kondyurin A.V., Leontyev V.B., Levkovich M.G., Mashinsky A.L., Nechitailo G.S. (2001). Polymerization in microgravity as a new process in space technology. Acta Astronaut..

[136] Pojman J., Ainsworth W., Chekanov Y., Masere J., Volpert V., Dumont T., Wilke H. (2000). Bubble behavior and convection in frontal polymerization on the KC-135 aircraft. Proceedings of the 38th Aerospace Sciences Meeting and Exhibit.

[137] Connell J.W., Crivello J.V., Bi D. (1995). Effect of low earth orbit atomic oxygen exposure on epoxy functionalized siloxanes. J. Appl. Polym. Sci..

